# Bimodal ^1^H Double Quantum Build-Up Curves by Fourier and Laplace-like Transforms on Aged Cross-Linked Natural Rubber

**DOI:** 10.3390/polym13203523

**Published:** 2021-10-13

**Authors:** Dumitrița Moldovan, Radu Fechete

**Affiliations:** Physics and Chemistry Department, Technical University of Cluj-Napoca, Memorandumului 28, R-400114 Cluj-Napoca, Romania

**Keywords:** natural rubber (NR), natural aged NR, DQ Fourier spectra, DQ Laplace-like spectra, distribution of ^1^H residual dipolar coupling in elastomers

## Abstract

The ^1^H DQ Fourier and Laplace-like spectra for a series of cross-linked natural rubber (NR) samples naturally aged during six years are presented and characterized. The DQ build-up curves of these samples present two peaks which cannot be described by classical functions. The DQ Fourier spectra can be obtained after a numeric procedure which introduces a correction time which depends less on the chosen approximation, spin-½ and isolated CH_2_ and CH_3_ functional groups. The DQ Fourier spectra are well described by the distributions of the residual dipolar coupling correlated with the distribution of the end-to-end vector of the polymer network, and with the second and fourth van Vleck moments. The deconvolution of DQ Fourier spectra with a sum of four Gaussian variates show that the center and the width of Gaussian functions increase linearly with the increase in the cross-link density. The Laplace-like spectra for the natural aged NR DQ build-up curves are presented. The centers of four Gaussian distributions obtained via both methods are consistent. The differences between the Fourier and Laplace-like spectra consist mainly of the spectral resolution in the favor of Laplace-like spectra. The last one was used to discuss the effect of natural aging for cross-linked NR.

## 1. Introduction

Homonuclear and heteronuclear residual dipolar couplings (RDCs) or quadrupolar interactions in soft solids such as elastomers and biological tissues represent an important source of information about the structure and molecular dynamics [1,2,3,4,5]. Proton residual dipolar couplings in elastomers reflect changes in the cross-link density, temperature, the uniaxial and biaxial extension or compression as well as the presence of fillers and penetrant molecules. Structure–dynamics–function relationships using RDCs can be investigated for the broad class of elastomer materials [2,3,4].

The measurements of RDCs can be carried out using one-dimensional (1D) and two-dimensional (2D) NMR methods. In the 1D case, methods were used such as the dipolar correlation effect in combination with Hahn and solid echo [6,7], the stimulated echo [8], the magic echo [9] and magnetization exchange [10,11]. Model free access to RDCs is given by the analysis of multiple-quantum (MQ) build-up [12,13,14,15,16,17,18] and decay [19] curves recorded in the initial regime of the excitation/reconversion periods of the experiment, as well as the accordion magic sandwich technique [20]. Chemically site-selective RDCs can be elucidated by 2D NMR spectroscopy using, for instance, ^13^C-^1^H heteronuclear residual dipolar couplings and encoded spinning sideband patterns [21]. The NOESY under magic angle sample spinning (MAS) [22] and double-quantum (DQ) MAS NMR spectroscopy are also used [23,24].

In general, residual dipolar couplings are characterized by heterogeneous distributions. In many cases, the local variation in cross-link density and the presence of network defects will lead to the variation of residual dipolar couplings around an averaged value. Furthermore, residual dipolar couplings and its associated dynamic order parameter are dependent on the time scale of the NMR experiment [2]. In such situations, the MQ build-up curves show a broad maximum from which the RDC distribution can be obtained by inverse Laplace transform by means of the Tikhonov regularization method [18], and references therein. A more complex situation is present when RDCs are affected, such as in the case of grafted polymer chains [14,25], the presence of fillers [26,27], radiation-induced cross-linking [15,28] or thermal degradation [29]. In these cases, bimodal DQ build-up curves were detected and the average RDCs distributions were analysed using DQ phenomenological growth curves [28,30].

In order to obtain magnetization relaxation times and diffusivities distributions, a fast algorithm of inverse Laplace transform (ILT) [31,32] from over almost two decades was applied successfully. Unfortunately, the ILT is ill-defined, sensitive to experimental noise and affected by numerical artifacts. In spite of these drawbacks, ILT was used for many 1D and 2D correlations and exchange experiments [33]. To the best of our knowledge, the advanced version of ILT was not applied for investigation of the multimodal MQ experiments.

Recently, S. Nie et al. [34] have studied cross-linked (natural rubber) NR with and without carbon black (CB) aged thermally by hot air or physically by mechanical fatigue at different time intervals. Their measurements are based on the nonlinear rheological parameter obtained from FT-Rheology and ^1^H DQ-NMR techniques. C. Huang et al. [35] combined ^1^H-DQ NMR techniques and the tube model (two different types of entanglements), i.e., transiently trapped entanglements (TTEs) and permanently trapped entanglements (PTEs). They obtained a new understanding of the architecture and composition of the network structure by studying the relationships between the varied performances at different testing conditions under deformation in unvulcanized NR. A.P. Munaro et al. [36] used ^1^H DQ and dipolar filtered magic sandwich echo (DF-MSE) methods to characterize the structural and dynamic modifications that occur during 12 years’ exposure to degrading harsh atmospheric conditions of insulator PDMS elastomeric networks.

The goal of this work is to characterize a series of cross-linked natural rubber (NR1-NR7) aged for six years in natural conditions for which a bimodal time domain DQ build-up curve was measured. The natural aging process on these cross-linked NR samples leads to a special behavior of the recorded DQ data, which cannot be considered a simplest superposition of two (or more) components. Therefore, the simplest classical models for analyzing the DQ experimental data fail, and new approaches have to be developed. These are based, not on the measurement of a limited number of parameters (average residual dipolar coupling, multi-spin van Vleck moments, etc.) which are globally characterizing the polymer network, but on the measurement of the distributions of residual dipolar coupling. For that, an automatic numeric procedure was developed based on Fourier transform into a spin-½ pair approximation. The distributions of residual dipolar coupling obtained by Fourier transform are compared with those obtained by Laplace-like inversion procedures which are assuming a spin-½ pair approximation or an ad hoc Abragam-like function as a kernel. Contrary to the classical methods which, in general, are using the data measured only in the initial time regime, the newest proposed procedures are applied on the entire DQ build-up curve.

## 2. Experimental

### 2.1. Samples

In this investigation, a series of cross-linked natural rubber samples aged by oxidative processes were used (Table 1). The investigated elastomer samples are from commercially available natural rubber (NR) SMR10 (Malaysia). The additives were 2 phr stearic acid and 3 phr (parts per hundred rubber) ZnO. The sulfur and accelerator contents are 1:5 phr each. The accelerator was TBBS (benzothiazyl-2-tert-butyl-sulfenamide). After mixing the compounds into a laboratory mixer at a temperature of 50 °C, the samples were vulcanized at 160 °C using a Monsanto MDR-2000-E vulcameter. The degree of cross-linking was measured after vulcanization at a temperature of 160 °C by the low- frequency shear modulus directly in the vulcameter. More about the sample characteristics of the series of NR were presented in previous papers [9,16,20,30,37,38,39]. The series of NR were manufactured in 2003. For the natural aging of the cross-linked NR rubber, the samples were simply stored in dark at room temperature for a period of several years. Then, from time to time, a series of normalized DQ build-up curves were recorded during this period of time for the same series of cross-linked natural rubber samples. These samples were cut off from the same 10 × 10 cm plate with the thickness of 3 mm.

### 2.2. NMR Measurements

The measurements, for natural aged samples, were performed using a BRUKER MINISPEC mq20 spectrometer working at 19.7 MHz. The sample temperature during all measurement was 35 °C. The DQ five-pulse sequence used to record the build-up curves is presented in Figure S1 (see Supplementary Information, where the efficiency in creating DQ coherences is also discussed). The tipping pulse length was 8–8.5 µs, dependent on the cross-link density. The excitation/evolution period is denoted by *τ* and was increased in equal steps up to 4 ms. The evolution period *t*_0_ and z-filter *t_z_* were kept short of the order of 20 and 50 μs, respectively. The recycle delay was 0.5 s. The double-quantum filtered signals were normalized to the integral intensity of the single-quantum signal measured in the same conditions as DQ build-up curves.

## 3. Proton DQ Build-Up Curves

**Figure 1:**
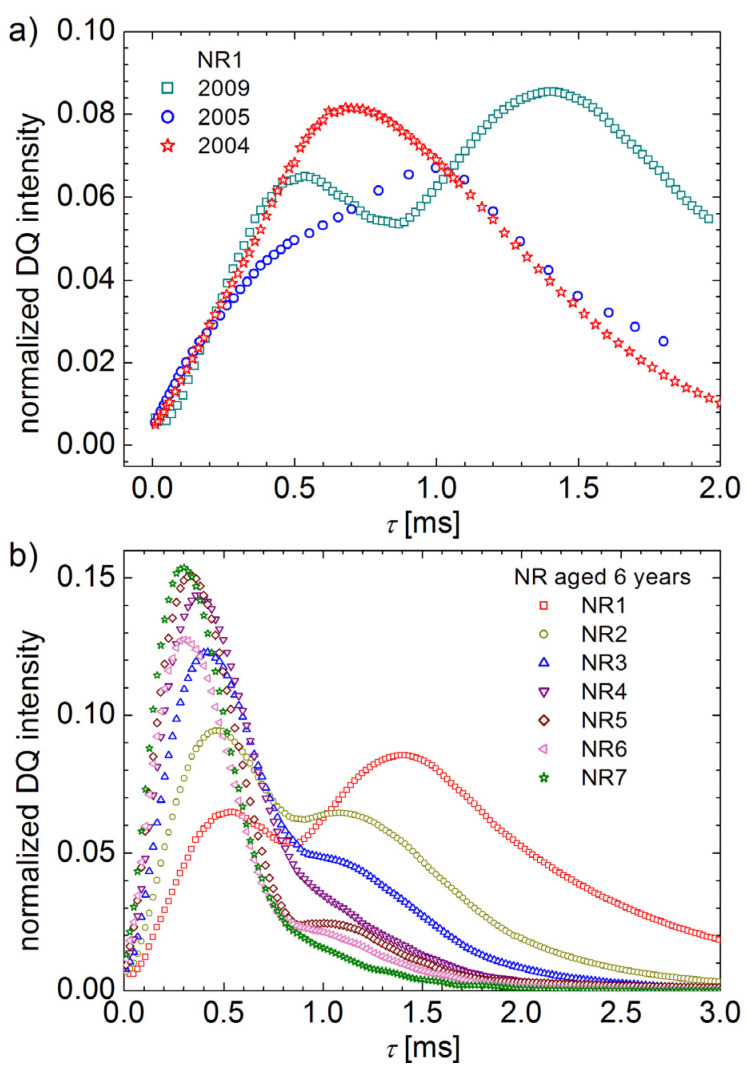
(**a**) Normalized ^1^H DQ build-up curve for natural aged cross-linked natural rubbers measured after one year, two years and six years from the production date. (**b**) ^1^H DQ build-up curves measured after six years after production as function of cross-link density (samples NR1 to NR7 from Table 1).

The DQ build-up curve was recorded from the unaged NR1 sample after the vulcanization (see Table 1) and is shown in Figure 1a. A single component can be observed with a maximum around 700 μs. The maximum DQ signal is obtained from the combined effect of an increase in the intensity of the DQ coherences and decay of the single-quantum coherences due to transverse relaxation during the excitation and reconversion periods of the pulse sequence.

The effect of one year of aging in natural conditions can be observed by the apparition of a new component in the DQ build-up curve as a shoulder shifted to larger time values compared with the maximum of unaged sample (Figure 1a). This new maximum can be associated with the apparition of polymer chain segments characterized by smaller residual dipolar couplings (RDCs) due to chain scissions by oxidative processes. The data obtained for small and large excitation/reconversion times are almost overlapping over the curve registered with one year before, but a drop is observed in the region of maximum that reflects the presence of two polymer networks with different RDCs. The additional five years of natural aging produces a large effect on the DQ curve. Two components are clearly observed, and the aging effect can be quantified as a displacement of a second component to longer excitation/reconversion times. This displacement can be associated with the increase in the transverse relaxation time and/or decrease in the residual dipolar interactions.

The second component is clearly observed for sample NR1, characterized by the smallest cross-link density, and is almost inexistent for sample NR7 (Table 1), characterized by the largest cross-link density (see Figure 1b). The effect of natural aging, revealed as multi-component ^1^H DQ build-up curves, decreases with the increase in the cross-link density.

## 4. The Multi-Spin van Vleck Moments Approximation

In many of the previous works, the DQ build-up curves were analyzed in the initial excitation/reconversion time regime where the measurements of the ^1^H residual dipolar couplings encoded the second van Vleck moments [16,19,30]. Since one cannot deduce an exact analytical expression to approximate the full DQ build-up and decay curve for a multi-spin system, the normalized DQ signal can be phenomenologically described in terms of the residual DQ second van Vleck moments ${\bar{M}}_{2}^{DQ}$ and effective relaxation times $T_{2}^{*}$, as [30]

(1) $$S_{DQ}\left(2\tau ,{\bar{M}}_{2}^{DQ},T_{2}^{*}\right)=\frac{1}{2}\left\{1-\exp\left(-\frac{1}{2}{\bar{M}}_{2}^{DQ}\tau^{2}\right)\right\}\exp\left(-\frac{2\tau }{T_{2}^{*}}\right)$$

where *τ* is the excitation/reconversion time periods and where the residual second van Vleck moment ${\bar{M}}_{2}^{DQ}$ is due to the spin-pair nature of the dipolar coupling Hamiltonian and to the fact that the DQ filter edits these pairs.

In Equation (1), the transverse relaxation process during free evolution is accounted by the exponential decay with an effective transverse relaxation time $T_{2}^{*}$. The Equation (1) is valid in the limits of $\tau \ll T_{2}^{*}$ and ${\left({\bar{M}}_{2}^{DQ}\right)}^{\frac{1}{2}}\tau \ll 1$.

In order to describe bimodal DQ curves, as obtained for the aged NR1 sample (Figure 1a), the theoretical expression could be a sum of two ad hoc functions as given by Equation (1)

(2) $$S_{DQ}\left(2\tau \right)=A_{1}\left[1-e^{-\frac{1}{2}{\bar{M}}_{2,1}^{DQ}\tau^{2}}\right]e^{-\frac{2\tau }{T_{2,1}^{*}}}+A_{2}\left[1-e^{-\frac{1}{2}{\bar{M}}_{2,2}^{DQ}\tau^{2}}\right]e^{-\frac{2\tau }{T_{2,2}^{*}}}$$

where the ${\bar{M}}_{2,1}^{DQ}$, ${\bar{M}}_{2,2}^{DQ}$, $T_{2,1}^{*}$ and $T_{2,2}^{*}$ are the residual DQ second order van Vleck moments and the effective transverse relaxation times of polymer chain segments characterized by different mobility. The *A*_1_ and *A*_2_ are two normalization constants where the quantity *A_i_*/(*A*_1_ + *A*_2_), (*i* = 1,2) indicates the proportion of two dynamic components.

**Figure 2:**
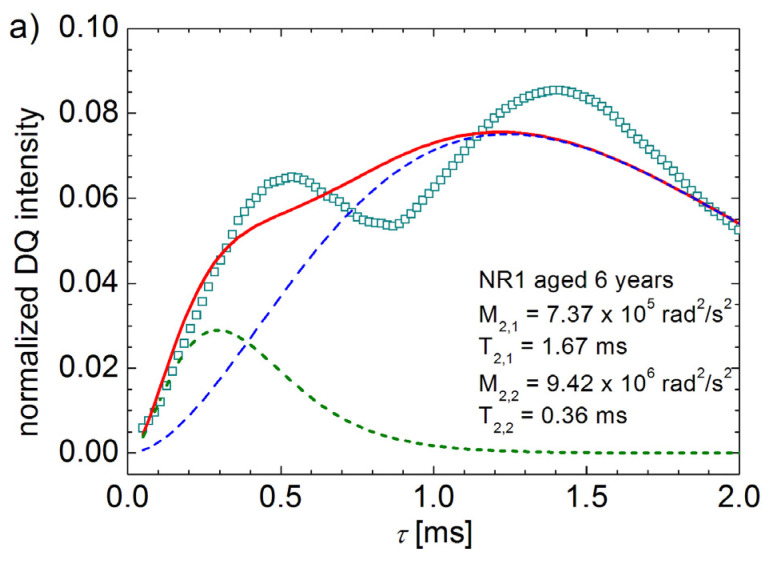

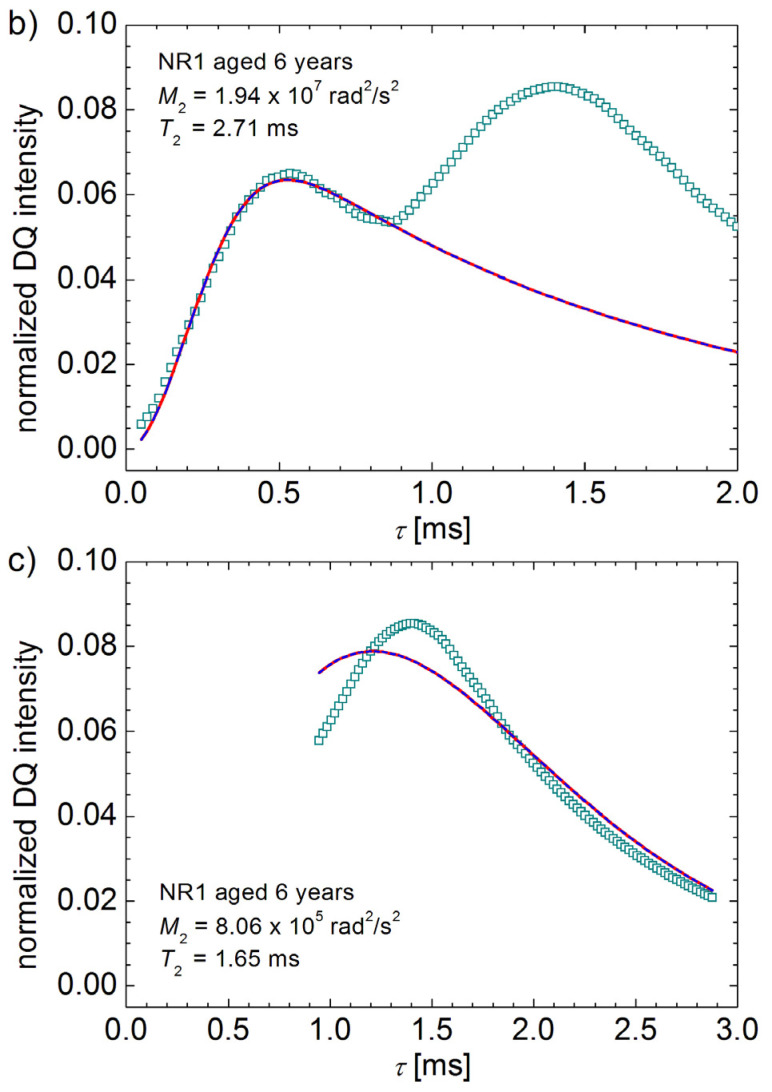
(**a**) The best fit of DQ build-up curve for sample NR1 aged six years using Equation (4). The best fit of the first peak (**b**) and the second peak (**c**) using Equation (2).

**Figure 3:**
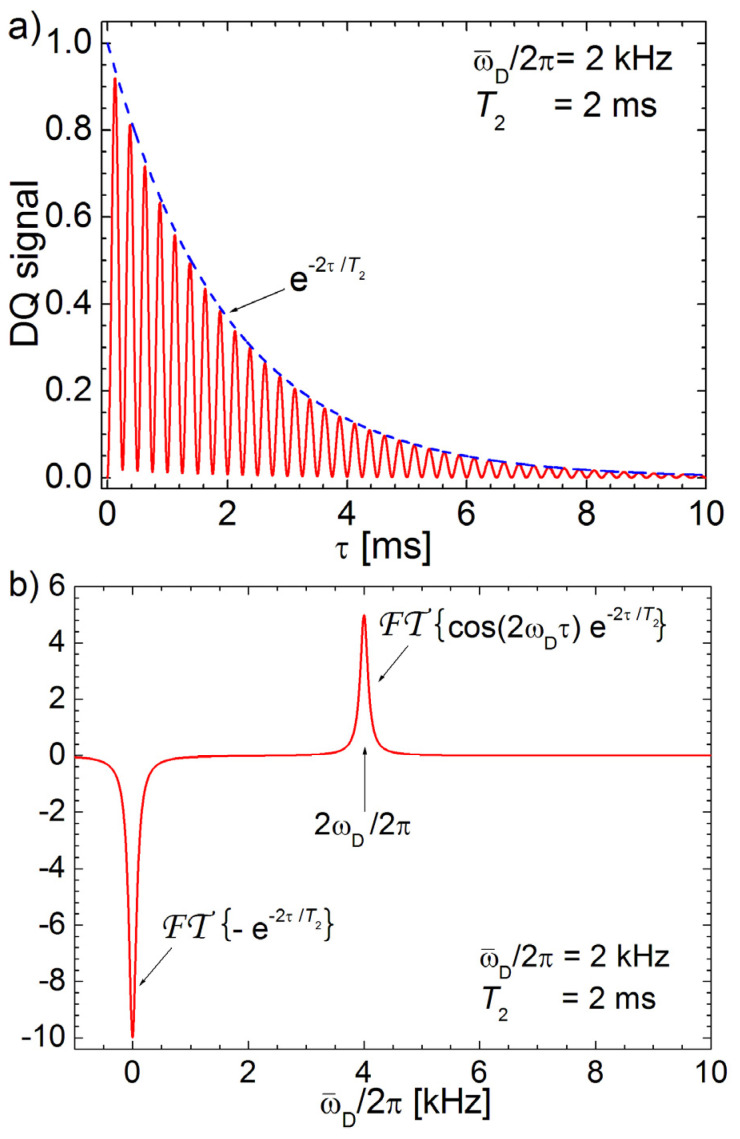

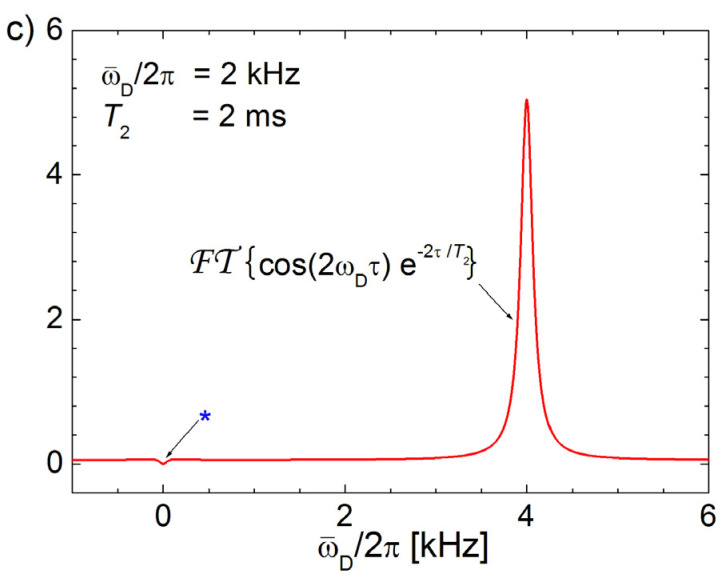
(**a**) Simulation of the normalized DQ build-up curve described by Equation (6) with a single residual dipolar coupling ${\bar{\omega }}_{D}/2\pi$ = 2 kHz and an effective relaxation time $T_{2}^{*}$ = 2 ms; (**b**) the Fourier spectrum of the DQ signal shown in (**a**) and (**c**) the corrected DQ Fourier spectra from (**b**). The star symbol showed the error in the transformed spectra due to the correction procedure.

Figure 2a shows the attempts to fit the experimental data using Equation (2) for NR1 sample six years aged. The failure is due to the fact that Equation (1) is not able to describe the experimental DQ build-up curves for the full excitation/excitation time regime. The best fit of the DQ build-up curve for the aged NR1 sample can approximate only the initial regime and fails dramatically for excitation/reconversion times larger than 0.3 ms (see Figure 3a). Moreover, the function described by Equation (2) as the superposition of two individual components, presented with dashed and short dashed lines in Figure 2a, is not showing a distinct double peak feature. As one can observe from this figure the smallest (left) peak can be approximated but for the large component (right) the theoretical peak (dashed line) is much broad. In fact, the fit of the first component can be conducted relatively well, as one can see from Figure 2b. For this fit, we consider only the experimental data up to ~0.85 ms of the beginning of the second built-up curve. Even in the absence of the small peak, if the behavior is described by Equation (1), the right peak cannot be approximated by any values of ${\bar{M}}_{2}^{DQ}$ and $T_{2}^{*}$ (Figure 2c).

## 5. The Distributions of Residual Dipolar Coupling Constants by Fourier Transform

### 5.1. The Spin-½ Pair Approximation

For a spin-½ pair all the terms of the Hamiltonian described by Equation (S1) in supplementary information) commute with each other, which allows obtaining an exact time evaluation of the spin system response to the DQ five pulse sequence that is given by

(3) $$S_{DQ}\left(2\tau \right)=\langle \sin^{2}\left({\bar{\omega }}_{D}\tau \right)\exp\left(-\frac{2\tau }{T_{2}^{*}}\right)\rangle$$

where ${\bar{\omega }}_{D}=\sqrt{3/2}{\bar{\omega }}_{D}$ and *τ* is the excitation/reconversion time period and $T_{2}^{*}$ is the effective transverse relaxation time of the single-quantum coherences. For simplicity, we can assume that the relaxation processes of the DQ coherences are characterized by the same value. In this section we will demonstrate that, due to the particular nature of the problem, this assumption will not significantly change the final result. The 〈(…)〉 represents the statistical average over the end-to-end vector, $\vec{R}$ and angle *β* between the direction of the pre-averaged end-to-end vector and external static magnetic field ${\vec{B}}_{0}$.

Figure 3a presents a simulation of a DQ signal (continuous line) for an ideal polymer characterized by a single value of the residual dipolar constant ${\bar{\nu }}_{D}={\bar{\omega }}_{D}/2\pi =2\;\mathrm{kHz}$ and an envelope of NMR signal (dashed line) characterized by the transverse relaxation time $T_{2}^{*}=2\;\mathrm{ms}$. We will use this simulated curve to demonstrate the simplest procedure based on the Fourier transform, (hereafter we use the notation FT {…}) to obtain the distribution of the residual dipolar couplings. The negative Fourier transform, −FT {…}, of the DQ filtered signal is presented in Figure 3b. The spectrum can be easily interpreted if we will rewrite the Equation (3)

(4) $$S_{DQ}\left(2\tau \right)=\langle \frac{1-\cos\left(2{\bar{\omega }}_{D}\tau \right)}{2}\exp\left(-\frac{2\tau }{T_{2}^{*}}\right)\rangle$$

The negative spectra obtained from Equation (4) can be written as a sum of two terms with the same weight:

(5) $$-𝓕𝓣\left\{S_{DQ}\left(2\tau \right)\right\}=-\frac{1}{2}𝓕𝓣\left\{\langle \exp\left(-\frac{2\tau }{T_{2}^{*}}\right)\rangle \right\}+\frac{1}{2}𝓕𝓣\left\{\langle \cos\left(2{\bar{\omega }}_{D}\tau \right)\exp\left(-\frac{2\tau }{T_{2}^{*}}\right)\rangle \right\}$$

i.e., a negative Lorentzian peak centered in origin (described by the first term) and a second positive Lorentzian peak centered at double value of residual dipolar coupling constant given by the last term in Equation (5). One can observe that, regardless of the value ${\bar{\omega }}_{D}/2\pi$, the first term is always negative centered in origin while the desired spectral amplitudes are disperse over all ${\bar{\omega }}_{D}/2\pi$ values. A simple numerical procedure can be designed to cancel the negative peak independent on the residual dipolar interactions. Due to the long wings of Lorentzian function the proper action is to be applied in the time domain instead on the frequency domain. Therefore, the Fourier transform procedure is applied on a new function defined in the time domain where an exponential decay with a correction time and amplitude ½ is added to the negative DQ signal

(6) $$\begin{array}{cc} 𝓕𝓣\left\{S_{\mathrm{DQ}}\left(2\tau \right),\;T_{2}^{*}\right\} & =𝓕𝓣\left\{-\langle \sin^{2}\left({\bar{\omega }}_{D}\tau \right)\exp\left(-\frac{2\tau }{T_{2}^{*}}\right)\rangle +\frac{1}{2}\exp\left(-\frac{2\tau }{\tau^{c}}\right)\right\}\\ & ≅\frac{1}{2}𝓕𝓣\left\{\langle \cos\left(2{\bar{\omega }}_{D}\tau \right)\exp\left(-\frac{2\tau }{T_{2}^{*}}\right)\rangle \right\} \end{array}$$

where $\tau^{c}$ is the correction time. This parameter has to be obtained from the best fit. The negative sign used before has the role to produce the final result into a form that is easily identifiable, by means of a positive Fourier spectral distribution of the residual dipolar constants.

A dedicated program was written in C++ to implement the steps presented in Equation (6). First, the correction time is chosen by the well-known secant method which systematically splits in half an interval described by two extreme $\tau^{c}$ values. For each particular $\tau^{c}$, a single point ${\left|𝓕𝓣\left\{\right\}\right|}_{{\bar{\omega }}_{D}=0}$ is considered until the best value is found

(7) $$\left|𝓕𝓣{\left\{S_{\mathrm{DQ}}\left(2\tau \right),\;T_{2}^{*}\right\}}_{{\bar{\omega }}_{D}=0}\right|\leq \varepsilon$$

where *ε* is a small positive constant which represents the chosen fit error. The final interval quantifies also the error in the determination of the correction time value $\tau^{c}$. The corrected spectrum of a doubled residual dipolar constant is presented in Figure 3c. The efficiency of the algorithm is demonstrated by the RDC distribution spectrum with a small residual contribution of around zero value marked by a star in Figure 3c. The Fourier transform with a correction time procedure was applied on the DQ build-up data normalized at the SQ amplitude for the entire NR sample series (see Figure 1b) and the corresponding normalized distributions of the residual dipolar couplings were obtained. The correction times $\tau^{c}$ are presented in Table 1.

### 5.2. The DQ Fourier Spectra

By applying the same steps presented for the spin-½ pair in Equations (3)–(6), we can extend the Fourier analysis, also as an approach to the cases of isolated CH_3_ functional groups. In this case, the spin system response on the DQ five pulse sequences has the same mathematical form as those presented in Equation (3), but the residual dipolar constant ${\bar{\omega }}_{D}^{{\mathrm{CH}}_{3}}$ is specific to the isolated CH_3_ functional group. From here, one can extend the approximation to the isolated CH_3_ and CH_2_ functional groups. The manner of treatment is unitary; the only thing which will be different is the prefactor of the exponential correction term from Equation (6)

(8) $$𝓕𝓣\left\{S_{\mathrm{DQ}}\left(2\tau \right)\right\}=\frac{1}{N}𝓕𝓣\left\{\sum_{i}A_{i}\sin^{2}\left({\bar{\omega }}_{D}^{\left(i\right)}\tau \right)\exp\left(-\frac{2\tau }{T_{2}^{\left(i\right)}}\right)+k\exp\left(-\frac{2\tau }{T_{2}^{\left(i\right)}}\right)\right\}$$

where *A*_i_ is the desired distribution function, and *N* and *k* are two constants specific to each approximation. Then, the corrected Fourier transform is:

(9) $$𝓕𝓣\left\{S_{\mathrm{DQ}}\left(2\tau \right)-k\exp\left(-\frac{2\tau }{\tau^{c}}\right)\right\}≅\frac{1}{N}FT\left\{-\sum_{i}A_{i}\cos\left(2{\bar{\omega }}_{D}^{\left(i\right)}\tau \right)\exp\left(-\frac{2\tau }{T_{2}^{\left(i\right)}}\right)\right\}$$

where *k* from Equation (9) is related to the *N* and *k* constants from Equation (8).

**Figure 4:**
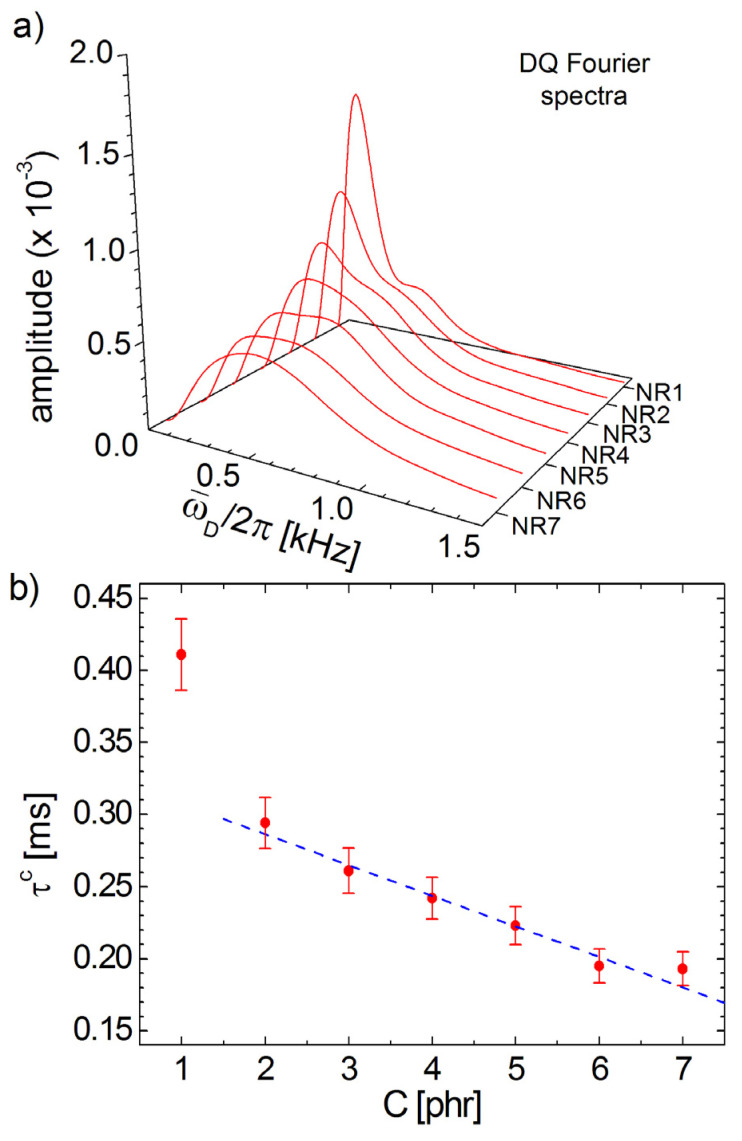
(**a**) Double-quantum Fourier spectra obtained from the DQ build-up curves the series of six-years aged cross-linked NR samples. (**b**) The dependence of the correction effective relaxation time functions of cross-link density. The dashed lines represent the linear fit of data for samples NR2 up to NR7.

Figure 4a presents the distributions of residual dipolar coupling as a corrected Fourier transform of normalized DQ build-up curves for the entire cross-linked series of NR samples while the Figure 4b presents the dependence of the $\tau^{c}$ on the cross-link density obtained using the Equation (9). The correction time $\tau^{c}$ characteristic to the NR series, with the exception of NR1, decays linearly with the increase in the cross-link density. Since all Fourier spectra are broadened due to the relaxation processes, the residual dipolar coupling distributions present two unresolved peaks (see Figure 4a). These two peaks are more evident for the NR1 sample and merge totally for the NR7 sample. With the increase in the cross-link density the distributions of ${\bar{\omega }}_{D}/2\pi$ becomes broader. At a simpler inspection, the residual dipolar coupling distributions look similar to those obtained in the case of spin-½ approximation, and this is the reason why the Fourier spectra are not presented for spin-½ approximation. In fact, in the following section, we will demonstrate the differences between the obtained spectra, considering the previous described approximations are so small that they can be neglected, compared with the differences between the DQ Fourier spectra obtained for different samples.

**Figure 5:**
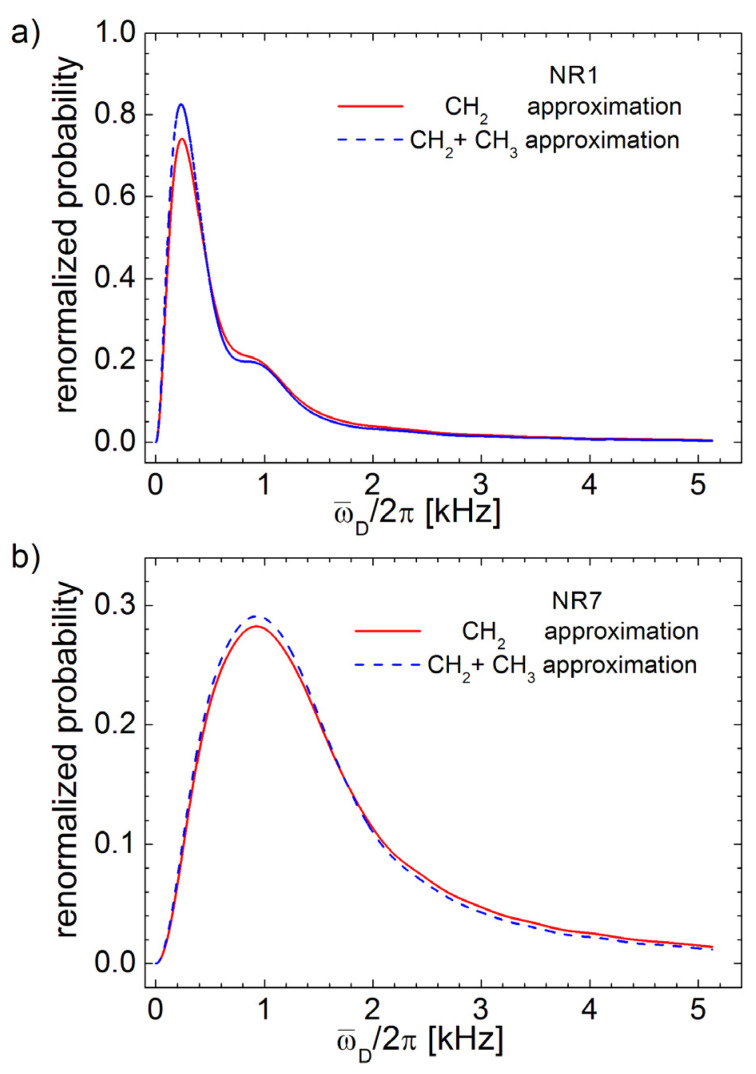
Comparison between DQ Fourier spectra obtained with spin-½ pair approximation and CH_2_ and CH_3_ isolated group’s approximation corresponding to (**a**) NR1 and (**b**) NR7-aged samples. In order to be compared, the Fourier spectra were renormalized to have the same integral area.

In order to be visually compared, in Figure 5 the distributions for both the spin-½ pair approximation (continuous black line) and for the isolated CH_3_ and CH_2_ functional groups approximation (dashed grey line) were represented together for the first and last series samples, which are NR1 and NR7. In order to evaluate the deviation of a curve from another curve, the merit value *χ*^2^ can be defined as

(10) $$\chi_{1,2}^{2}=\frac{\sum_{i=1}^{N}{\left[y_{1}^{\left(i\right)}-y_{2}^{\left(i\right)}\right]}^{2}}{N}$$

where *y*_1,2_ are the amplitude values of arbitrary (1) and (2) distribution curves. Then, it will be interesting to evaluate the error obtained in the case of bad choice of the approximation compared with the differences between two distributions obtained for samples with successive value of the cross-link density. For example, the merit value *χ*^2^ for the NR1 Fourier spectra obtained for spin-½ pair approximation and with isolated CH_3_ and CH_2_ functional groups approximation is $\chi_{\mathrm{NR}1}^{2}/\chi_{\mathrm{NR}1,\mathrm{NR}2}^{2}≅5.4\%$ from the merit value *χ*^2^ obtained by comparing the NR1 with NR2 Fourier spectra. This percentage of the merit value increases at $\chi_{\mathrm{NR}6}^{2}/\chi_{\mathrm{NR}6,\mathrm{NR}7}^{2}≅30.1\%$ for NR6 Fourier spectra compared with the differences between NR6 and NR7 spectra. In conclusion, since the differences between any two samples are much largest than the error due to the bad choice of the model, the Fourier spectra of a series of aged cross-linked natural rubber samples can be well characterized by any model, presented earlier as approximations.

### 5.3. The Distributions of End-to-End Distance and Residual Dipolar Coupling

The Fourier spectra of DQ curves for the aged natural rubber present the general features observed earlier for the distribution of residual dipolar coupling described in references [15,17,40]. As in the case of a 2 wt.% PDMS, see ref. [40], our DQ Fourier spectra consist of a high-narrow peak at lower residual dipolar coupling values and a tail which decays slowly with the increase in the residual dipolar coupling values. This resemblance between our Fourier spectra and the distribution of the residual dipolar coupling obtained from FTIKREG based on Tikhonov regularization [15] and then a fit [41,42,43] allow us to assume that the Fourier spectra features are mainly due to the distribution of the residual dipolar couplings and the relaxation times *T*_2_ will just broaden the obtained spectra. Therefore, in the following sections we characterize the DQ Fourier spectra for the cross-linked natural rubber samples in terms of the distribution of the residual dipolar couplings related to the distribution of the end-to-end vector of the polymer chains.

The 3D Gaussian probability distribution of the end-to-end vector, $\vec{R}$ for a cross-linked polymer network, is given by

(11) $$P_{3D}\left(\vec{R}\right)={\left(\frac{3}{2\pi \langle R^{2}\rangle }\right)}^{3/2}\exp\left(-\frac{3{\vec{R}}^{2}}{2\langle R^{2}\rangle }\right)$$

where $\langle R^{2}\rangle$ is the average square of the end-to-end distance and the distribution over the length of the end-to-end vector, *R*, which satisfies the normalization relation

(12) $$\int_{0}^{\infty }4\pi R^{2}P_{3D}\left(R\right)dR=1$$

The Gaussian distribution of the length of the end-to-end vector can be written as

(13) $$P_{G}\left(R\right)=4\pi R^{2}P_{3D}\left(R\right)dR=4\pi R^{2}{\left(\frac{3}{2\pi \langle R^{2}\rangle }\right)}^{3/2}\exp\left(-\frac{3R^{2}}{2\langle R^{2}\rangle }\right)dR$$

From Equations (11)–(13), the corresponding distribution of the residual dipolar coupling constant is given by the Г function [15,17]:

(14) $$P_{\Gamma }\left({\bar{\omega }}_{D}\right)=\frac{2}{\sqrt{\pi }}\sqrt{\frac{27}{8}\frac{{\bar{\omega }}_{D}}{{\langle {\bar{\omega }}_{D}\rangle }^{3}}}\exp\left(-\frac{3}{2}\frac{{\bar{\omega }}_{D}}{\langle {\bar{\omega }}_{D}\rangle }\right)$$

where ${\bar{\omega }}_{D}$ is the residual dipolar coupling and $\langle {\bar{\omega }}_{D}\rangle$ is the mean residual dipolar coupling. This distribution fast increases around ${\bar{\omega }}_{D}/2\pi =0$ and then slowly after a maximum decay with a long tail at large ${\bar{\omega }}_{D}/2\pi$. By its nature, the Г distribution is a broad one; it does not consider the powder average (see simulations from supplementary information) and therefore cannot explain the narrow peak observed at lower ${\bar{\omega }}_{D}$ values. This narrow peak corresponds to a Gaussian distribution of residual dipolar coupling, which is defined as

(15) $$P_{Gauss}\left({\bar{\omega }}_{D}\right)={\left(\frac{3}{2\pi \cdot \Delta {\bar{\omega }}_{D}{}^{2}}\right)}^{1/2}\exp\left(-\frac{3{\left({\bar{\omega }}_{D}-{\bar{\omega }}_{D}^{0}\right)}^{2}}{2\cdot \Delta {\bar{\omega }}_{D}{}^{2}}\right)$$

where ${\bar{\omega }}_{D}^{0}$ is the maximum value of the Gaussian distribution and $\Delta {\bar{\omega }}_{D}$ is the width of Gaussian function. This function leads to a distribution of the end-to-end vector of the form

(16) $$P_{Gauss}\left(R\right)dR=\frac{2}{\pi }\sqrt{\frac{3}{2}}\frac{R}{\langle R^{2}\rangle }\exp\left(-\frac{3{\left(R^{2}-R_{0}^{2}\right)}^{2}}{2{\langle R^{2}\rangle }^{2}}\right)dR$$

In conclusion, the distribution of residual dipolar coupling may be described by the superposition of Г function and Gaussian function described by Equations (14) and (15), respectively. More details about the mediation over azimuthally angle *β* and end-to-end vector $\vec{R}$ can be found in the Supplementary Information.

### 5.4. The Characterization of DQ Fourier Spectra

**Figure 6:**
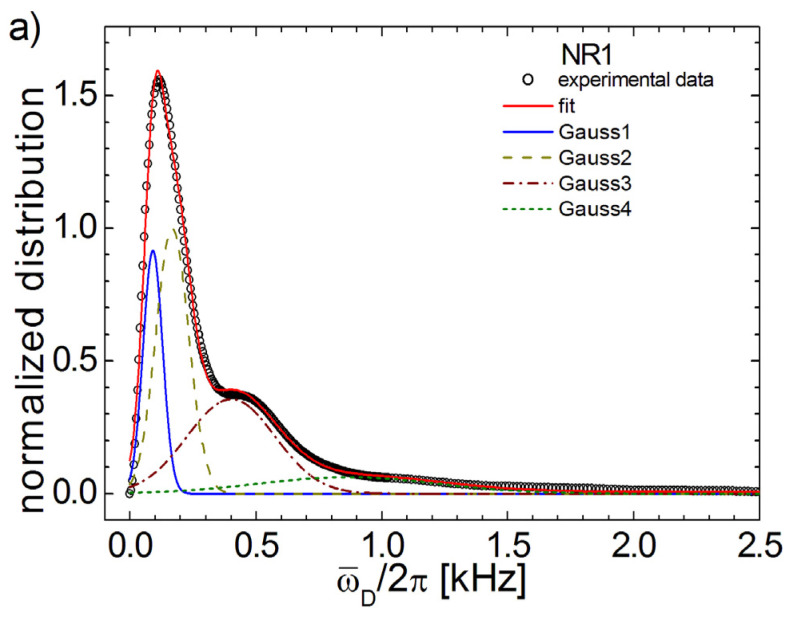

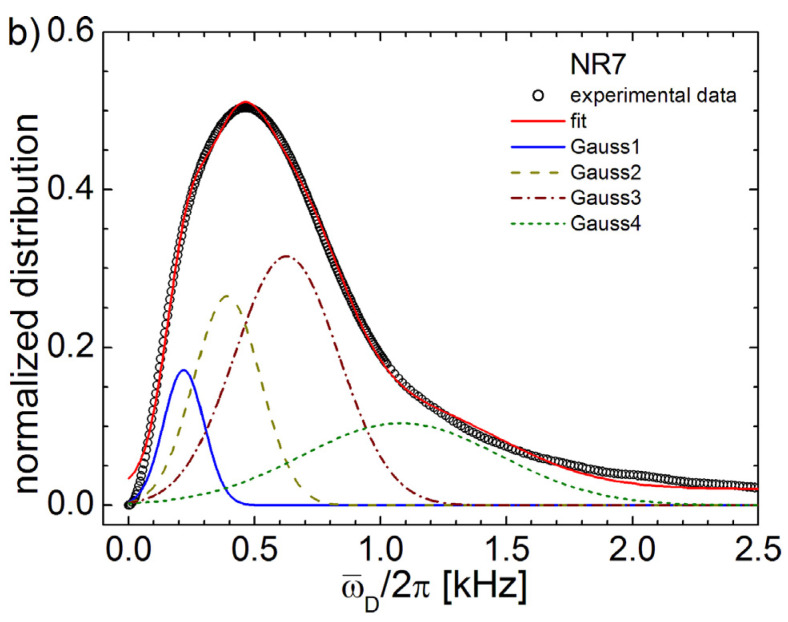
The deconvolution fit (continuous line) of DQ Fourier spectra (open circles) of six-years aged NR1 (**a**) and NR7 (**b**) cross-linked natural rubber. The best fits were obtained with a sum of four Gaussian functions described by Equation (16) and are shown with continuous, dashed, dash-dot and small-dash lines.

As discussed earlier, the DQ Fourier spectra of aged cross-linked natural rubber consist of two components. The fits of theses Fourier spectra with a sum of Г and Gaussian functions for each component leads to unsatisfied results. In fact, even by increasing the number of components, the presence of the Г function leads to unsatisfied fits of the DQ Fourier spectra. The best fit (continuous line) was found when the DQ Fourier spectrum (open circles) was deconvoluted with four Gaussian functions (continuous line, dashed line, dash-dot line and small dash line). The deconvolution of ^1^H DQ Fourier spectra for the aged NR1 and NR7 are presented in Figure 6. The deconvolution of the DQ Fourier spectra corresponding to the aged NR1 sample approximate the spectra well while some inconsistencies can be observed for NR7. From these deconvolutions, one can observe that there are two Gaussian distribution (continuous and dashed lines in Figure 6) responsible for the fit of the peak located at small residual dipolar coupling values, and two Gaussian distribution (dash-dot line and small dash line) responsible for the fit of the small peak located at larger residual coupling values.

The DQ Fourier spectra can be characterized by the residual van Vleck moments. The second and fourth van Vleck moment for the DQ Fourier spectra obtained with the approximation of isolated CH_2_ and CH_3_ functional groups for the entire series of aged NR samples are presented in Table 1. The second *M*_2_ and fourth *M*_4_ residual van Vleck moments present a monotone variation with the increase in the cross-link density, with the exception of NR5. This is due to the fact that the van Vleck moment is calculated from DQ Fourier spectra after the determination of spectral maximum position ${\bar{\omega }}_{D}^{max}/2\pi$, presented in Table 1 in the last column. The DQ Fourier spectra of aged NR samples consist of two compo-nents; therefore, they are asymmetrical. From sample NR1 to sample NR4, the maximum was found for the first peak, which is characterized by small values of the residual dipolar coupling. Starting with NR5, the maximum jumps towards the second peak, which affects the dependence of the of the *M*_2_ and *M*_4_ van Vleck moments of the DQ Fourier spectra function of cross-link density. The second *M*_2_ and fourth *M*_4_ residual van Vleck moments for all Gaussian distributions obtained from deconvolution of the DQ Fourier spectra for the entire series of aged natural rubber samples are listed in Table 2.

### 5.5. The Effect of Cross-Link Density

**Figure 7:**
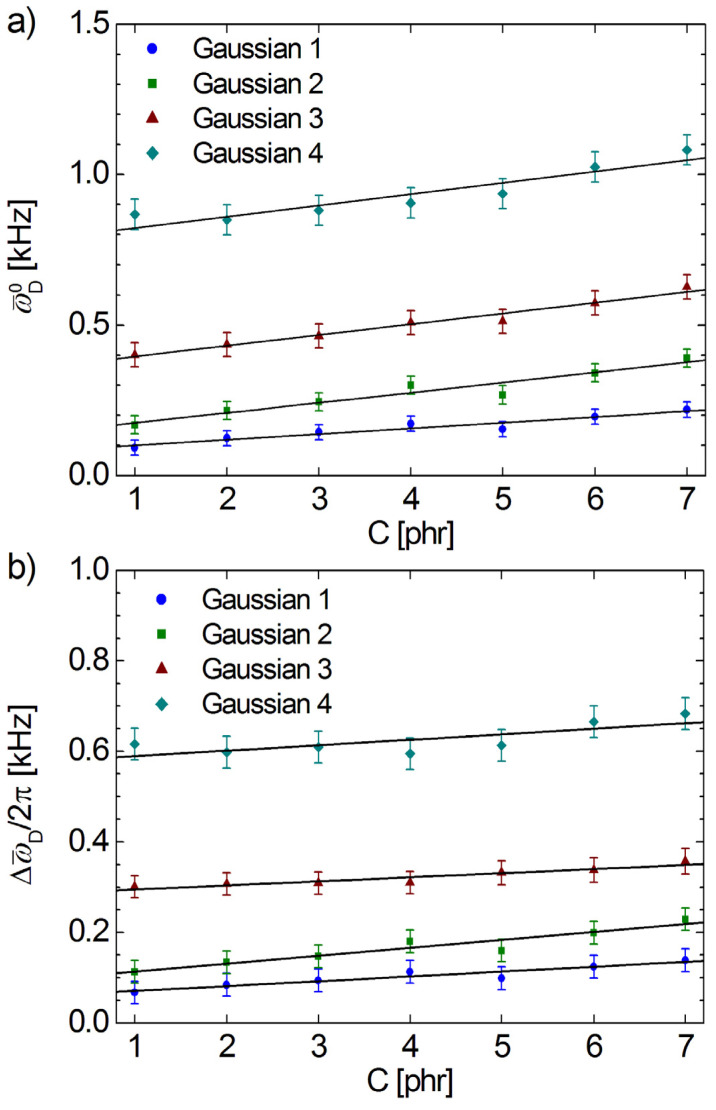
The dependence of (**a**) the mean value of the residual dipolar couplings and (**b**) line width for the Gaussian distribution functions of the cross-link density C (sulfur accelerator content—phr). In all cases, the lines represent the best fit of the data.

The Gaussian distribution (14) is characterized by the center of distribution ${\bar{\omega }}_{D}^{0}$, where one can find the maximum probability and by the width of residual dipolar coupling distribution $\langle {\bar{\omega }}_{D}\rangle$ (see Table 3). Linear dependences for all of these parameters which describe the distribution of residual dipolar couplings with the cross-link density were found (see Figure 7). The effect of an increase in the cross-link density was to proportionally increase the mean residual dipolar coupling for Gaussian distributions (see Figure 7a). At the same time, the increase in the number of cross-linking points leads to a larger inhomogeneity of the polymer network, which is observed in Figure 7b from the increase in the distribution width.

## 6. The Distributions of Residual Dipolar Couplings by Laplace-like

### 6.1. The Laplace-like Analysis of Bimodal ^1^H DQ Build-Up Curves

**Figure 8:**
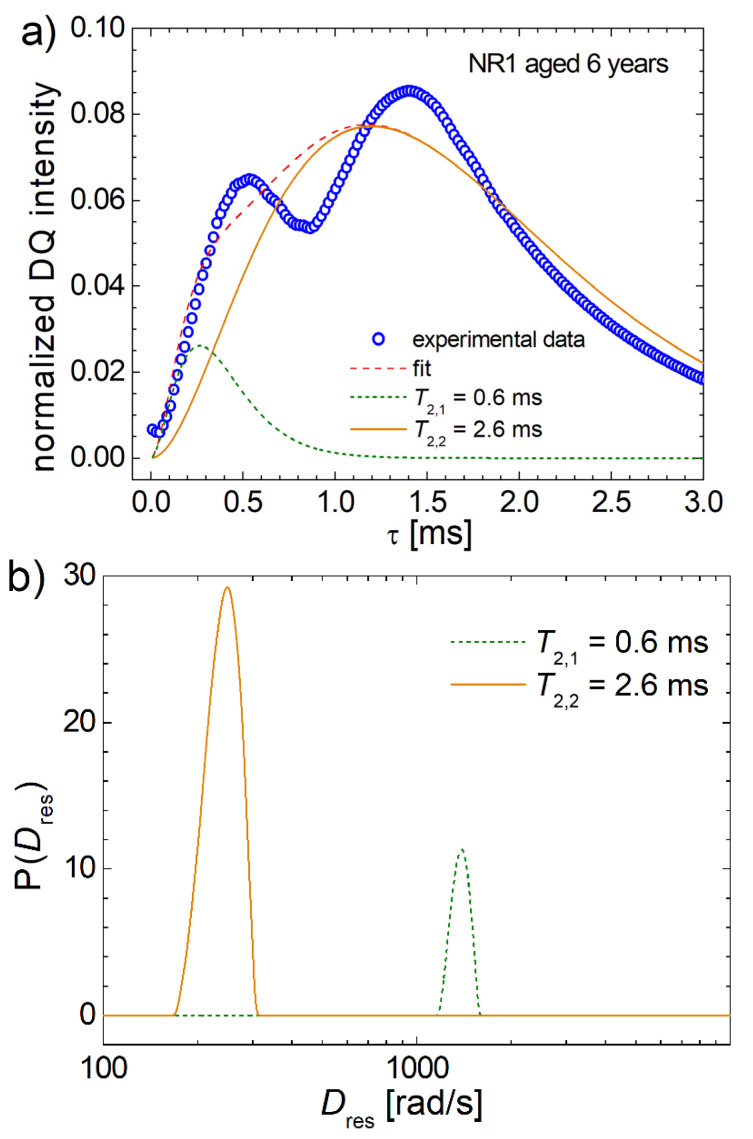
(**a**) The DQ build-up curve (open circle) measured for the NR1 sample naturally aged during 6 years, the fitting curve obtained after the Laplace-like inversion (dashed line) using a full mediation over average over dimensionless squared end-to-end vector, ${\vec{q}}^{2}$ and azimuthal angle β (Equation (S11) from Supplementary Information), the DQ NMR signals corresponding to the small *D*_res_ values (continuous orange line) and to the large *D*_res_ values (dotted olive line); (**b**) the distributions of residual dipolar coupling constants resulted from the analysis of data presented in (**a**) by Laplace-like inversion using Equation (S11) with $T_{2,1}^{*}=0.6\;\mathrm{ms}$ and $T_{2,2}^{*}=2.6\;\mathrm{ms}$ obtained as the best fit of experimental data.

If we will take a look at the final expressions of DQ signals in Equation (4), we reach the conclusion that the differences between the mentioned approximations are small. Therefore, in any of the previous approximations we must analyze a signal of the form

(17) $$S_{DQ}\left(2\tau ,T_{2}^{*}\right)=\exp\left(-\frac{2\tau }{T_{2}^{*}}\right)\int_{0}^{\infty }\int_{0}^{\pi }g\left(R\right)\cdot h\left(\beta \right)\sin^{2}\left[{\bar{\omega }}_{D}\left(R,\beta \right)\cdot \tau \right]d\beta \cdot dR$$

and since ${\bar{\omega }}_{D}\left(R,\beta \right)$ is a function of *R* and *β*, this can be rewritten as

(18) $$S_{DQ}\left(2\tau ,T_{2}^{*}\right)=\exp\left(-\frac{2\tau }{T_{2}^{*}}\right)\int_{0}^{\infty }f\left({\bar{\omega }}_{D}\right)\sin^{2}\left({\bar{\omega }}_{D}\tau \right)d{\bar{\omega }}_{D}$$

where we assumed an effective averaged transverse relaxation time. A complete mediation over end-to-end vector $\vec{R}$ or/and azimuthally angle *β*, and the distribution of the residual dipolar coupling constant *D*_res_, is presented in the Supplementary Information with many details. The best fit of the DQ build-up curve measured for natural NR1 aged during six years of analyses by Laplace-like inversion using Equation (S12) with the kernel presented in SI.13 (see Supplementary Information) and with the best transverse relaxation times $T_{2,1}^{*}=0.6\;\mathrm{ms}$ and $T_{2,2}^{*}=2.6\;\mathrm{ms}$, respectively, is presented in Figure 8a. Unfortunately, the best fit curve (dashed red line) cannot explain the bimodal character of the measured data for six years of naturally aged NR1. The overlap of the two signals originating from a large residual dipolar coupling constant *D*_res_ (olive dotted line) characterized by $T_{2,1}^{*}=0.6\;\mathrm{ms}$ and from a small residual dipolar coupling constant *D*_res_ (olive dotted line) characterized by $T_{2,2}^{*}=2.6\;\mathrm{ms}$ present just a small shoulder at the initial time regime. This fit is similarly with that presented in Figure 2a and analyzed in terms of second van Vleck moments ${\bar{M}}_{2}^{DQ}$ analyzed with Equation (4). The distributions of the small and large values of residual dipolar coupling constant *D*_res_ are presented in Figure 8b. Slightly asymmetrically, these distributions are similarly with those reported in several papers for the residual dipolar coupling constant *D*_res_ [41,42]. Then, the complete mediation over $\vec{R}$ and *β* was proved not to be able to describe the measured bimodal DQ build-up curve for natural aged NR samples, but could be used to describe a bimodal DQ build-up curve for a non-aged PDMS1 sample [25] (for more details, see the Supplementary Information).

As we mentioned before, the ^1^H DQ Fourier spectra are affected by the effective transverse relaxation time. Another method, which can provide us with a good resolution in the distribution of residual dipolar coupling, can be based on the Laplace inversion [26,27,31,32,39,44]. In fact, we must underline that the true Laplace inversion is characterized by an exponential kernel specific to magnetization relaxation processes, i.e.,

(19) $$M\left(t\right)=\int_{0}^{\infty }f\left(T_{2}\right)e^{-\frac{t}{T_{2}}}dt$$

The quantity of interest is the distribution function *f*(*T*_2_). This can be obtained using the fast inversion algorithm which assumes a problem written into a matrix form as [31,32]

(20) M=K⋅F+E

where the matrix *M* contain the measured data, *K* is the kernel, *E* stores the measurement noise and F is the desired distribution. We have adapted the problem by changing the exponential integrand kernel from Equation (19) into an $sin^{2}\left({\bar{\omega }}_{D}\tau \right)$ as in Equation (18), and which can be solved using the FMI (*F*ast *M*atrix *I*nversion) algorithm. In this case, hereafter the inversion problem will be called a Laplace-like problem. Additionally, we must implement a method to obtain the effective transverse relaxation time, $T_{2}^{*}$. For that, we have considered a minimum $T_{2,min}^{*}$ and a maximum $T_{2,max}^{*}$ value for effective transverse relaxation time, and for values between these two limits we tested the fit of the experimental data, or more specifically the merit function $\chi_{}^{2}$ as it is described by Equation (10).

**Figure 9:**
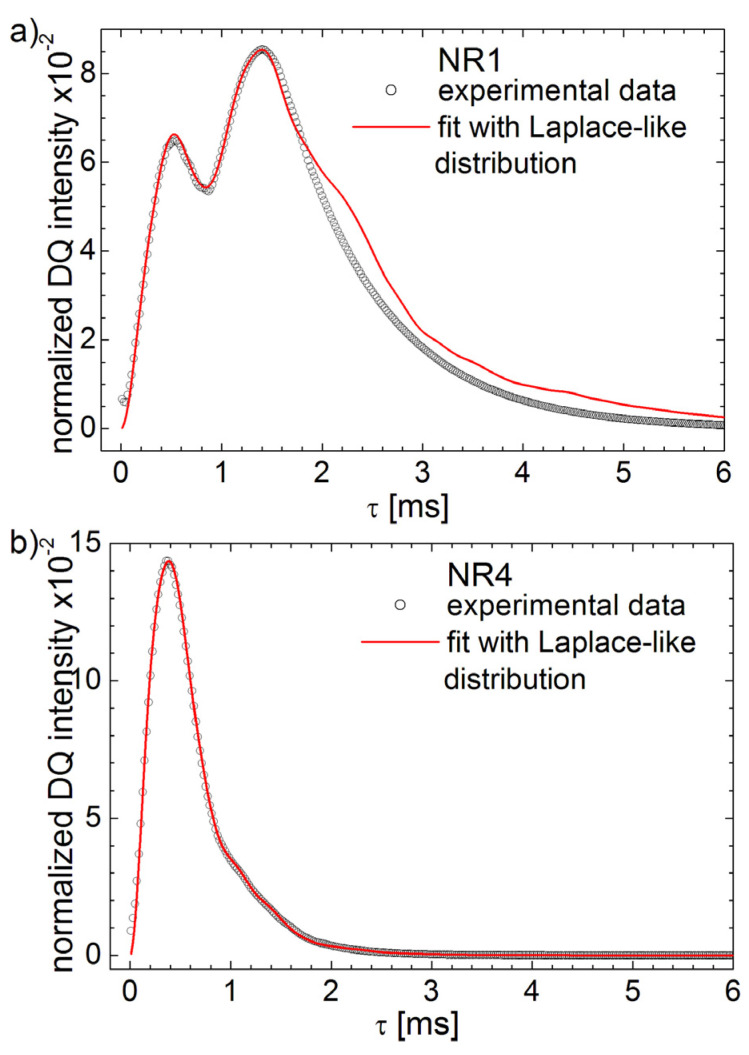

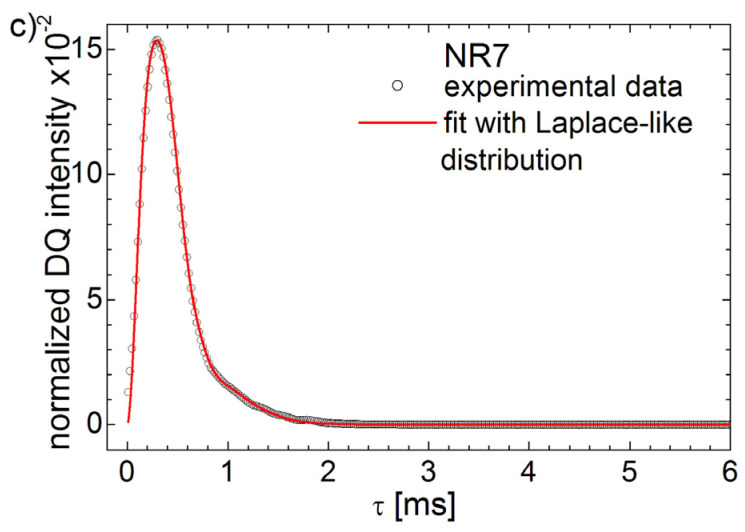
The experimental ^1^H DQ build-up curves (open circles) and fits (continuous line) using the Laplace-like inversion procedure for (**a**) NR1, (**b**) NR4 and (**c**) NR7 cross-linked natural rubber six years natural aged.

The experimental ^1^H DQ build-up curves and fits with Laplace-like inversion procedure are presented in Figure 9 for natural aged samples NR1, NR4 and NR7. The inverse Laplace-like procedure can fit well the ^1^H DQ build-up curve recorded for the aged NR1 sample up to the maximum (*τ* ≅ 1.4 ms), but there are some deviations after this maximum (see Figure 9a). Nevertheless, this is a much better fit compared with the approximation obtained before with multi-moments method (see Figure 2), described in Section 3, since now both build-up components are fitted. The deviation observed at larger *τ* values is due to the fact that the special kernel used in this case $sin^{2}\left({\bar{\omega }}_{D}\tau \right)$ is periodical and we assumed a single effective relaxation time $T_{2}^{*}$.

A better approximation of the measured ^1^H DQ build-up curves are observed for the naturally aged rubber samples with higher cross-link density (see Figure 9b,c). In these cases, the inverse Laplace-like procedure can fit well the entire experimental ^1^H DQ build-up curves. Some extremely small oscillations can be observed due to the periodicity of the used kernel and probably due to the fact that the second component in the distribution of RDCs associated with the aging effect is much smallest in these cases.

### 6.2. Proton DQ Laplace-like Spectra of Aged Natural Rubber

**Figure 10:**
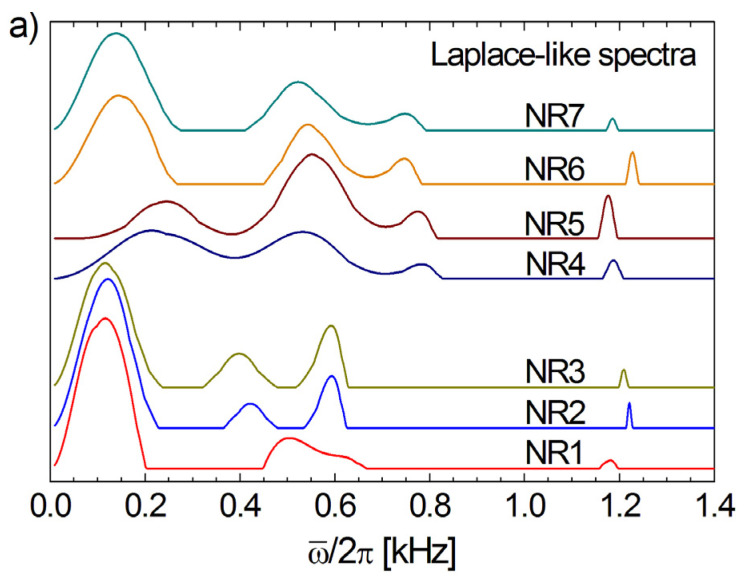

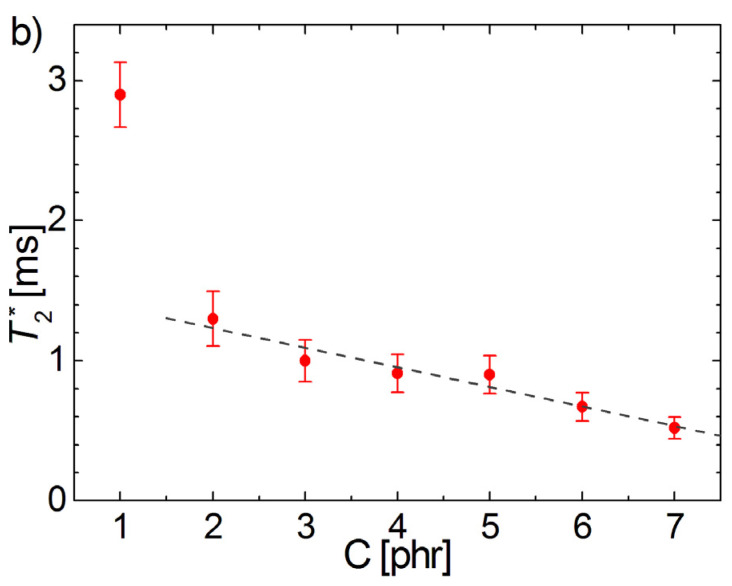
(**a**) The inverse Laplace-like spectra (Equation (18)) which show the distributions of residual dipolar couplings ${\bar{\omega }}_{D}/2\pi$ for the series of cross-linked natural rubber naturally aged for six years. (**b**) The effective transverse relaxation time as a function of cross-link density obtained from the best fit of the normalized ^1^H DQ build-up curves using the inverse Laplace-like transformation. The dashed line represents the best liner fit of the data for the samples NR2 to NR7.

The normalized distributions $f\left({\bar{\omega }}_{D}\right)$ of the residual dipolar couplings, or with other words, the ^1^H DQ Laplace-like spectra, are presented in Figure 10a for all naturally aged rubber samples. All ^1^H DQ Laplace-like spectra consist of four well-resolved peaks with one exception: the middle peaks for the aged NR1 sample. For small and large values of cross-link density, the main peak is characterized by a reduced value of averaged residual dipolar couplings (${\bar{\omega }}_{D}/2\pi =100-250\;\mathrm{Hz}$). From this point of view, the samples NR4 and NR5 are not in the range and present the first peak at ${\bar{\omega }}_{D}/2\pi =200-250\;\mathrm{Hz}$. This is not a surprising result, since in some of our previous results [19,39], the NR4 sample was also showing a different behavior for various measured microscopic NMR parameters and elasticity modulus.

The best-fitted effective relaxation times $T_{2}^{*}$ for all aged NR1–NR7 samples are presented in Figure 10b as a function of cross-link density. As in the case of correction time obtained by ^1^H DQ Fourier spectra (see Figure 4), a linear dependence of $T_{2}^{*}$ as a function of cross-link density can be observed, with the exception of the NR1 sample. From our observation, the value of $T_{2}^{*}$ can have a certain influence on the ^1^H DQ Laplace-like spectra (Figure 10a), but in the fitting error limit (see Figure 10b) this can be negligible compared with the differences between DQ Laplace-like spectra.

**Figure 11:**
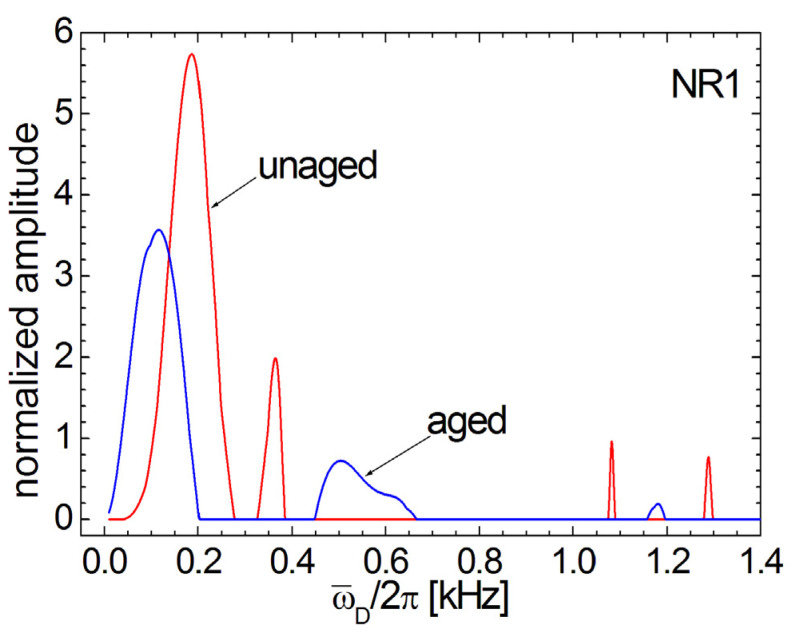
Comparison between ^1^H DQ Laplace-like spectra for the six-years aged (gray line) and unaged (black line) NR1 natural rubber sample. The distributions were renormalized in order to have the same integral area.

Figure 11 presents a comparison between the normalized ^1^H DQ Laplace-like spectra corresponding to aged and unaged NR1 samples. Additionally, the unaged ^1^H DQ Laplace-like spectrum is composed of four peaks but with a different distribution. Two large distribution peaks are located at lower residual dipolar coupling values (${\bar{\omega }}_{D}/2\pi =200-400\;\mathrm{Hz}$), and two smallest peaks are located at larger residual dipolar coupling values (${\bar{\omega }}_{D}/2\pi \approx 1.075-1.3\;\mathrm{kHz}$ ). The peaks located at lower residual dipolar coupling values are in agreement with the results obtained by Nie et al. [34] and with the distributions reported in [35,36,45]. After an aging in natural conditions, many of the NR1 polymer chains characterized by a small value of ${\bar{\omega }}_{D}/2\pi$ become more mobile and have a reduced residual dipolar coupling (see the left large peak). The polymer chains with a small value of ${\bar{\omega }}_{D}/2\pi$ together with the polymer chain with ${\bar{\omega }}_{D}/2\pi \approx 350\;\mathrm{Hz}$ become more rigid at ${\bar{\omega }}_{D}/2\pi =\approx 450-650\;\mathrm{Hz}$. After aging, the two peaks located at larger residual dipolar coupling values collapse into a small peak located between them at ${\bar{\omega }}_{D}/2\pi \approx 1175\;\mathrm{Hz}$. It is evident from Figure 11 that the aging process induces a much broader heterogeneity in RDC and is characterized by larger mobility of polymer segments.

### 6.3. Proton DQ Fourier and DQ Laplace-Like Spectra of Aged Natural Rubber

**Figure 12:**
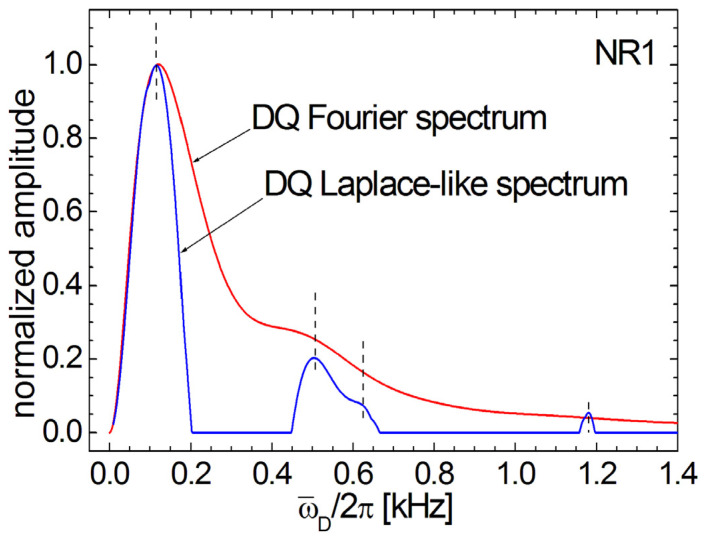
The comparison of ^1^H DQ Fourier and DQ Laplace-like spectra for the NR1 natural rubber sample, natural ages during six years are shown. The two spectra were renormalized in order to have the maximum value equal to one. The vertical dashed lines were drawn to cross the Fourier and Laplace-like spectra for the position of the local maxima for the four peaks corresponding to Laplace-like distribution.

The ^1^H DQ Fourier and DQ Laplace-like spectra can be directly compared (Figure 12) for the sample NR1, which is most affected by aging in natural condition. For this purpose, both spectra were renormalized, having the maximum amplitude 1 obtained at almost the same residual dipolar coupling value ${\bar{\omega }}_{D}/2\pi \approx 100\;\mathrm{Hz}$ (see also the left vertical dashed line in Figure 12). Up to the maximum value, one can observe an excellent superposition of both DQ Fourier and Laplace-like spectra, then the DQ Laplace-like peak decays faster. The second group of two joined peaks at median values of ${\bar{\omega }}_{D}/2\pi$ in the ^1^H DQ Laplace-like spectra can have a correspondent (see the second and third vertical dashed lines in Figure 12) with a shoulder in the ^1^H DQ Fourier spectra, but the four and smallest peak from DQ Laplace-like spectra hardly can be associated with some shoulder in the DQ Fourier spectra. It is obvious from Figure 6 and Figure 12 that the deconvolution of ^1^H DQ Fourier spectra, with four Gaussian functions, can not be so well matched with the peak distributions from ^1^H DQ Laplace-like spectra. Moreover, the peaks maximum from DQ Laplace-like spectra will not have a linear dependence function of cross-link density.

## 7. Comparison between Fourier and Laplace-like Methods

The ^1^H DQ Fourier and Laplace-like spectra were applied to analyze a series of bimodal DQ build-up curves characteristic to aged samples since the classical methods fail to produce acceptable results. Nevertheless, these methods also have some disadvantages: the main criticism of ^1^H DQ Fourier analysis is that the obtained spectrum is affected by the relaxation processes, which broadens the individual lines; therefore a supplementary deconvolution has to be applied. For both methods, we have to consider first a model in order to calculate the DQ signal function of excitation/reconversion times and then an automatic correction procedure has to be applied to obtain the ^1^H DQ Fourier spectra and another procedure must be applied to calculate the effective relaxation time to obtain the ^1^H DQ Laplace-like spectra. The Fourier analysis is well defined, which means that with the exception of a coefficient ~ ½ in front of the exponential correction term, which cancels the negative peak, the Fourier spectra are uniquely defined. The procedure for Laplace-like inversion deal with an ill conditioned problem and the results depend on various internal parameters. Moreover, since in our case the particular kernel is periodic, special attention has to be paid to the upper limits of the residual dipolar coupling values. Another disadvantage of the use of ^1^H DQ Fourier and Laplace-like analysis is the long measurement times. In order to obtain a DQ Fourier spectrum with no *wiggles*, due to the truncation of experimental data, we had to measure many points at large excitation/reconversion time *τ*. Despite all these, we found that the ^1^H DQ Fourier and Laplace-like analysis give complementary results and can be used successfully to analyze multi-component DQ build-up curves, such as those recorded for the cross-linked natural rubber aged in natural conditions over six years.

## 8. The ad Hoc Abragam-like Function for the Distribution of Residual Dipolar Coupling Constant *D*_res_

In recent years, an empirical function, so-called *Abragam-like*, became preferred to be used to describe the DQ build-up curve on isotropic samples [41,42]. Moreover, K. Saalvächter and co. state that the *Abragam-like* build-up curve can be used in the fitting of any kind of DQ build-up data for homogeneous or inhomogeneous samples [41]. Then, in this case, the DQ signal can be described as

(21) $$S_{DQ}\left(2\tau ,T_{2}^{*}\right)=\exp\left(-\frac{2\tau }{T_{2}^{*}}\right)\overset{\infty }{\underset{0}{\int }}P\left(D_{res}^{A-l}\right)K\left(D_{res}^{A-l},\tau \right)dD_{res}^{A-l}\;$$

where the distribution function was labeled with *P* ($D_{res}^{A-l}$), where $D_{res}^{A-l}$ can be viewed similar to a second-moment-type quantity and the specific kernel is given by [41,42]

(22) $$K\left(D_{res}^{A-l},\tau \right)=\frac{1}{2}\left\{1-\exp\left[-{\left(0.378D_{res}^{A-l}\;\tau \right)}^{1.5}\right]\right\}\times \cos\left(0.583\;D_{res}^{A-l}\;\tau \right)$$

The prefactors of *D*_res_ and the Weibull coefficient were considered from ref. [42,43]. In general, these coefficient and exponential factors can be optimized by fitting the DQ build-up curve with the Kernel (Equation (22)) multiplied with the exponent that represents the transverse relaxation process during the excitation and reconversion of double-quantum coherences.

**Figure 13:**
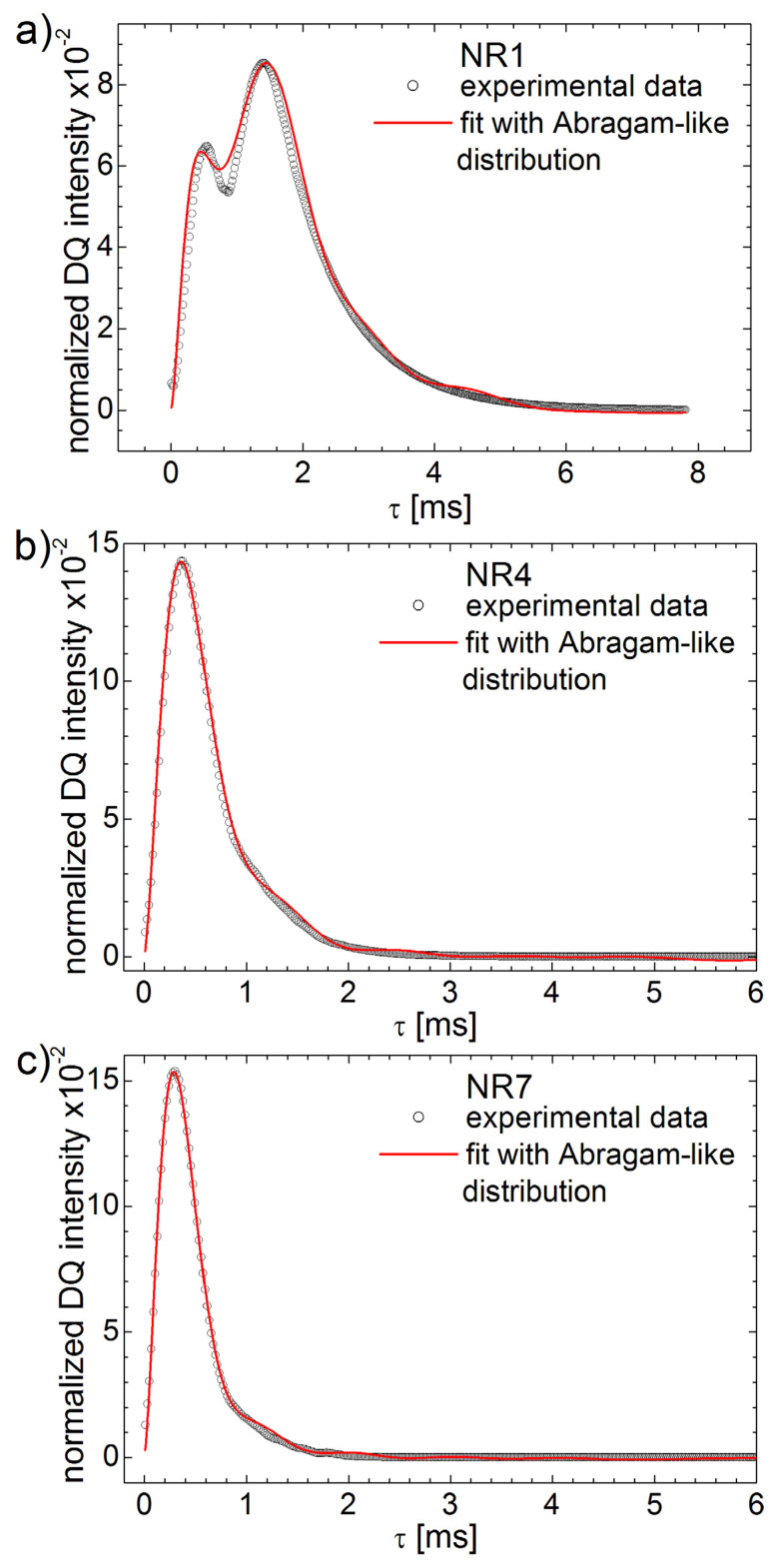
The experimental ^1^H DQ build-up curves (open circles) and fits (continuous line) using the Abragam-like inversion procedure (Equation (21) with the kernel given by Equation (22)) for (**a**) NR1; (**b**) NR4 and (**c**) NR7 cross-linked natural rubber aged naturally for six years.

The best fits of the experimental DQ build-up curves obtained using Equation (21) with the *Abragam-like* kernel are presented in Figure 13 for NR1, NR4 and NR7 natural rubber samples aged for six years. In all cases, one can remark a good fit of the experimental data. Among these, as expected, the largest value of the merit function (see Equation (10)) was obtained for NR1. This is due mostly to the fact that the experimental data are not so well approximated in the initial time regime up to *τ* ≅ 1 ms. With the increase in cross-link density, the effect of aging is reduced and the DQ build-up curves are better approximated on the entire time scale (see Figure 13b,c).

**Figure 14:**
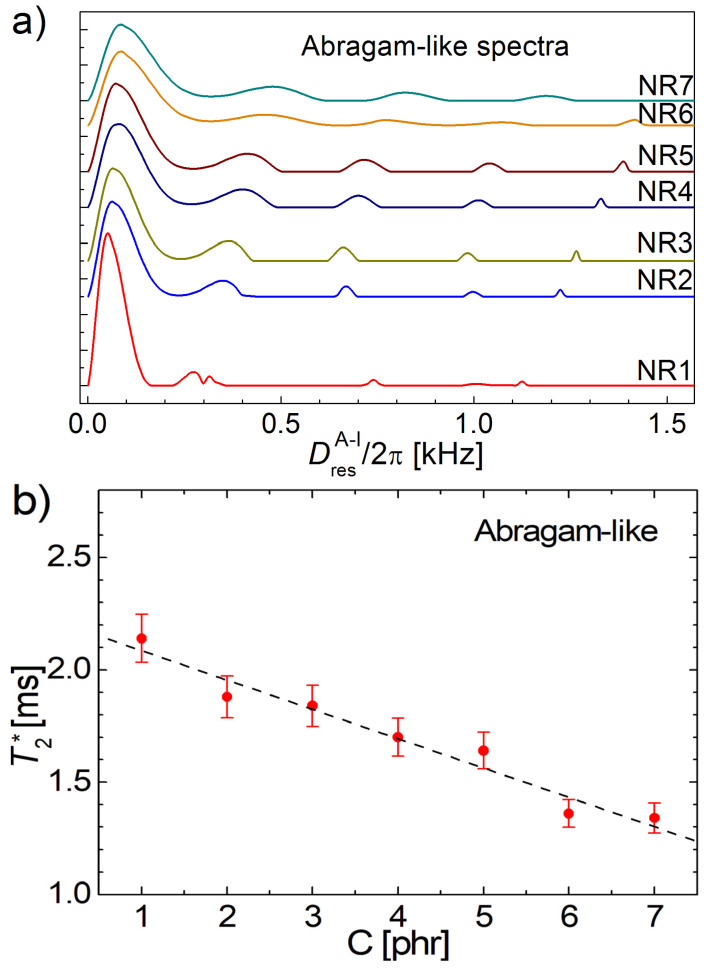
(**a**) The inverse Abragam-like spectra (Equation (21)) with the kernel Equation (22), showing the distributions of $D_{res}^{A-l}/2\pi$ for the series of six year natural aged of the cross-linked natural rubber. (**b**) The effective transverse relaxation time as a function of cross-link density obtained from the best fit of the normalized ^1^H DQ build-up curves using the inverse Abragam-like transformation. The dashed line represents the best line of fit of the data for the samples NR1 to NR7.

In Figure 14a, the distributions of $D_{res}^{A-l}$ for the six-year-aged cross-linked natural rubber series (NR1-NR7) obtained using Equation (21) with the *Abragam-like* kernel (Equation (22)) are presented. Compared with the series of distributions of the residual dipolar couplings ${\bar{\omega }}_{D}$ (shown in Figure 7a), in this case: (i) the main peak (with the largest integral area) is located at low $D_{res}^{A-l}/2\pi$ value (~53.2 Hz for NR1 to ~86.1 for NR7); (ii) a series of four (an additional one) peaks are observed at large $D_{res}^{A-l}/2\pi$ value all with a more small amplitude; (iii) the variation of $D_{res}^{A-l}$-distribution in function of cross-link density is more smooth than in the case of ${\bar{\omega }}_{D}$-distribution. Regarding the number of components and the particular domain, the obtained distribution of $D_{res}^{A-l}/2\pi$ is similar to those reported by Chassé et al. in ref. [42] for a mixture of NR–C2 samples. In the experimental error limit, the variation of the effective transverse relaxation time $T_{2}^{*}\;$, with cross-link density, decay linearly for the entire series NR1 to NR7 (see Figure 14b).

**Figure 15:**
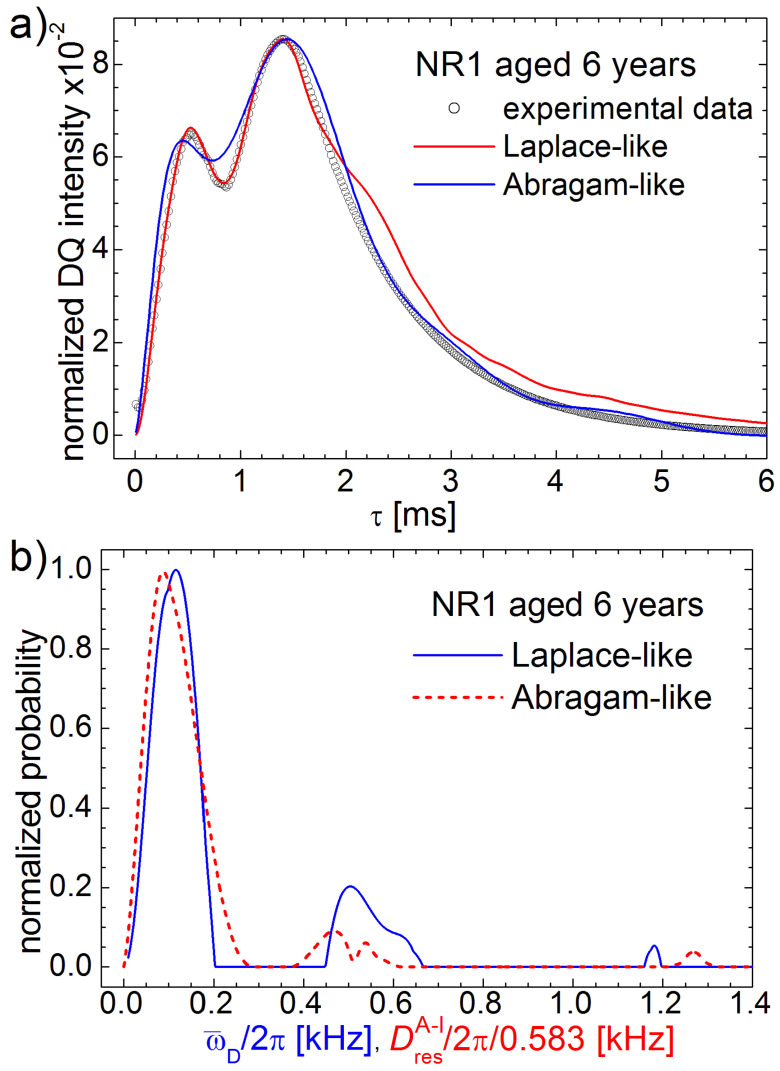
The comparison of DQ Laplace-like (continuous blue line) and Abragam-like (dashed red line) of (**a**) DQ build-up curve and (**b**) spectra measured for the NR1 natural rubber sample, naturally aged during six years. The spectra were renormalized in order to have the maximum value equal to one. Laplace-like spectrum is represented in function of averaged residual dipolar coupling, ${\bar{\omega }}_{D}/2\pi$ while the Abragam-like spectrum is represented in function of $D_{res}^{A-l}/2\pi /0.583$.

Comparing the best fit of DQ build-up curves measured for aged NR1 and analyzed spin-½ pair approximation (blue continuous line in Figure 15a) where the curves are well approximated in the initial time regime, inclusive of the maximum doublet, in the case of an *Abragam-like* kernel (red dashed line in Figure 15a), the experimental data are much better approximated for a medium and large excitation/reconversion time, *τ*, but not so well in the region of the maximum doublet. Due to this fact, one can consider that the spin-½ pair approximation (leading to so-called Laplace spectrum) is more precise in order to describe the effects of natural aging of natural rubber on the measured bimodal DQ build-up curve than in the case of the use of the Abragam-kernel. Nevertheless, one can expect similarly results. In order to test this hypothesis, the so-called Laplace-like and so-called *Abragam-like* distributions are represented together in Figure 15b for the NR1 natural sample aged for six years. The spectra are renormalized so that the maximum amplitude is 1. The *Abragam-like* distributions are rescaled with a factor of 0.583 present in Equation (22), where the *Abragam-like* kernel was defined. In the presented range, both distributions can be considered similarly.

## 9. Conclusions

The DQ build-up curves for a series of cross-linked natural rubber aged in natural conditions for six years were characterized by ^1^H DQ Fourier and Laplace-like spectra. For that, a numerical program was written in C++ to perform a correction with an effective relaxation time, which allows us to reveal the spectral distributions of the residual dipolar couplings. The ^1^H DQ Fourier spectra was treated in terms of a superposition of four Gaussian distributions of the residual dipolar coupling. The parameters which are obtained as a result of correction with an effective relaxation time and as result of spectral deconvolution seem to depend linearly on the cross-link density. The same measured ^1^H DQ build-up curves were used to obtain the ^1^H DQ Laplace-like spectra for the aged natural rubber samples. Four resolved Gaussian-like peaks were obtained in the ^1^H DQ Laplace-like spectra. Three methods (based on Fourier analysis, spin-½ pair approximation and on the ad hoc Abragam-like kernel) were presented to successfully fit the DQ build-up curves affected by six years of natural aging of a series of cross-linked natural rubber, whereas the classical ones fail. Finally, the aging effects on the dynamics of the NR polymer chain were discussed, and we find out that the aging of NR in natural conditions will increase the mobility of the majority of mobile polymer segments. At the same time, while aging, part of these polymer chains will become more rigid.

## Figures and Tables

**Table 1:** The values for the correction time $\tau^{c}$ obtained as a result of correction procedure of the DQ Fourier spectra based on spin-½ and isolated CH_2_ and CH_3_ approximations for the series of cross-link density from NR1 to NR7. The second *M*_2_ and fourth *M*_4_ residual van Vleck moments and the maximum of the DQ Fourier spectra obtained for the last approximation.

Sample	Sulfur Accelerator (phr)	$\tau^{c}$ Isolated-½ Spin (µs)	$\tau^{c}$ Isolated CH_2_ and CH_3_ (µs)	*M*_2_ [10^4^ rad^2^ s^−2^]	*M*_4_ [10^10^ rad^4^ s^−4^]	${\bar{\omega }}_{D}^{\max}/2\pi$ [Hz]
NR1	1–1	340.9	411	2.78	1.51	116.9
NR2	2–2	243.3	294	3.66	2.01	167.5
NR3	3–3	216.5	261	4.00	2.14	207.3
NR4	4–4	201.0	242	3.88	1.99	290.3
NR5	5–5	185.0	223	3.08	1.32	488.4
NR6	6–6	162.3	183	3.80	1.79	450.4
NR7	7–7	151.8	196	3.73	1.71	464.1

Fit errors were smallest than 5%.

**Table 2:** The second *M*_2_ and fourth *M*_4_ residual van Vleck moments specific to the Gaussian distributions obtained from the deconvolution of DQ Fourier spectra.

Sample	Gauss 1		Gauss 2		Gauss 3		Gauss 4	
	*M*_2_ 10^2^ [rad^2^ s^−2^]	*M*_4_ 10^5^ [rad^4^ s^−4^]	*M*_2_ 10^2^ [rad^2^ s^−2^]	*M*_4_ 10^6^ [rad^4^ s^−4^]	*M*_2_ 10^3^ [rad^2^ s^−2^]	*M*_4_ 10^8^ [rad^4^ s^−4^]	*M*_2_ 10^3^ [rad^2^ s^−2^]	*M*_4_ 10^9^ [rad^4^ s^−4^]
NR1	1.24	4.88	6.65	7.71	4.32	3.42	6.61	2.25
NR2	1.41	9.10	6.83	11.50	5.20	4.41	8.55	2.74
NR3	1.36	11.00	6.25	12.80	5.95	5.20	10.3	3.46
NR4	1.78	20.9	9.28	28.60	5.57	5.04	11.10	3.60
NR5	0.71	6.37	4.69	11.40	7.50	7.61	13.20	4.68
NR6	1.85	26.5	8.75	33.30	6.27	6.82	15.50	6.33
NR7	2.15	38.7	15.10	75.5	6.82	8.36	15.50	6.71

Fit errors were smallest than 5%.

**Table 3:** The centers ${\bar{\omega }}_{D}^{0}/2\pi$ and widths $\langle {\bar{\omega }}_{D}/2\pi \rangle$ of the Gaussian distributions obtained from the deconvolution of DQ Fourier spectra obtained for aged NR samples.

Sample	Gauss 1		Gauss 2		Gauss 3		Gauss 4	
	${\bar{\omega }}_{D}^{0}/2\pi$ [Hz]	$\Delta {\bar{\omega }}_{D}/2\pi$ [Hz]	${\bar{\omega }}_{D}^{0}/2\pi$ [Hz]	$\Delta {\bar{\omega }}_{D}/2\pi$ [Hz]	${\bar{\omega }}_{D}^{0}/2\pi$ [Hz]	$\Delta {\bar{\omega }}_{D}/2\pi$ [Hz]	${\bar{\omega }}_{D}^{0}/2\pi$ [Hz]	$\Delta {\bar{\omega }}_{D}/2\pi$ [Hz]
NR1	92.3	67.1	168.7	113.1	401.3	301.0	866.9	615.8
NR2	124.6	84.2	215.4	134.1	435.2	307.4	849.1	597.7
NR3	144.6	94.1	245.2	147.1	464.1	309.5	880.4	609.4
NR4	171.9	112.9	300.1	180.3	508.9	310.2	905.5	594.7
NR5	153.8	98.9	268.3	159.8	512.9	332.1	936.1	612.9
NR6	195.4	124.2	341.2	199.7	573.6	338.3	1025.2	665.2
NR7	218.8	138.8	390.8	229.1	626.7	357.5	1081.5	683.1

Fit errors were smallest than 5%.

## Data Availability

Data sharing not applicable.

## Supplementary Materials

The following are available online at https://www.mdpi.com/article/10.3390/polym13203523/s1, Figure S1: (a) General set-up of a double quantum (DQ) NMR experiment; (b) five pulses DQ experiment and (c) seven pulses DQ experiment with two refocusing pulses in the middle of excitation and reconversion periods; Figure S2: Double quantum (DQ) build-up curves measured for a 6 years natural aged NR1 sample using the 5 pulse DQ sequence (open red square) and 7 pulse DQ sequence (open blue circle); Figure S3: (a) The simulated bar plot distribution of ${\bar{\omega }}_{D}$ given by the Equation (S10) for constant ${\vec{q}}^{2}D_{\mathrm{res}}$ considering an isotope angular dependence described by sin function of azimuthal angle β shown in the small inclusion; (b) the comparison between the positive simulated bar plot distribution of ${\bar{\omega }}_{D}$ from Figure S3 (a) and a specific powder spectrum; Figure S4: The simulated bar plot distribution of ${\bar{\omega }}_{D}$ given by the Equation (S10) for constant $D_{\mathrm{res}}P_{2}\left\{\cos\left(\beta \right)\right\}$ considering only the distribution of dimensionless squared end-to-end vector; Figure S5: The simulated bar plot distribution of ${\bar{\omega }}_{D}$ given by the Equation (S10) for constant $D_{\mathrm{res}}$ for a combined isotope angular dependence described by sin function of azimuthal angle β and (a) a 3D Gaussian distribution of the end-to-end vector and (b) an Gaussian distribution of dimensionless squared end-to-end vector with the center $\left|{\vec{q}}_{0}\right|=2.5$; Figure S6: (a) Simulated bar plot distributions of residual dipolar coupling constant *P*($D_{\mathrm{res}}$) for a: (I) mono-Gaussian with $D_{\mathrm{res}}^{0}=500\;\mathrm{rad}/s$; (II) mono-Gaussian with $D_{\mathrm{res}}^{0}=2000\;\mathrm{rad}/s$ and (III) bi-Gaussian with $D_{\mathrm{res},1}^{0}=500\;\mathrm{rad}/s$ and $D_{\mathrm{res},2}^{0}=2000\;\mathrm{rad}/s$. (b) The simulated DQ build-up curves using Equation (S12) and kernel (SI.13) for the simulated distributions presented in a) and $T_{2}^{*}=2\;\mathrm{ms}$; Figure S7: (a) Simulated bar plot bi-Gaussian distributions of residual dipolar coupling *P*($D_{\mathrm{res}}$) with $D_{\mathrm{res},1}^{0}=500\;\mathrm{rad}/s$ and $D_{\mathrm{res},2}^{0}=5000\;\mathrm{rad}/s$ and the resulting residual dipolar coupling constant distribution *P*($D_{\mathrm{res}}$) (continuous line) after Laplace-like analysis based on Equation (S14) with $T_{2,1}^{*}=2\;\mathrm{ms}$ and $T_{2,2}^{*}=0.2\;\mathrm{ms}$. (b) The simulated DQ build-up curve (open circles) using Equation (S14) and the fitting of simulated DQ build-up data (continuous line); Figure S8: (a) The DQ-build-up curve (open circle) similarly with those measured by Bertmer et al. in [24] for a PDMS1 sample, the fitting curve obtained after the Laplace-like inversion using Equation (S13) (dashed line), the DQ NMR signals corresponding to the small *D*_res_ values (continuous orange line) and to the large *D*_res_ values (dotted olive line); (b) The distributions of residual dipolar coupling constants resulted from the analysis of data presented in (a) by Laplace-like inversion using Equation (S14) with $T_{2,1}^{*}=0.22\;\mathrm{ms}$ and $T_{2,2}^{*}=3.92\;\mathrm{ms}$.

## References

[1] Addad J.P.C. (1993). NMR and fractal properties of polymeric liquids and gels. Prog. NMR Spectrosc..

[2] Asano A. (2015). NMR Relaxation Studies of Elastomers. Annu. Rep. NMR Spectrosc..

[3] Demco D.E., Hafner S., Spiess H.W. (2002). Handbook of Spectroscopy of Rubbery Materials.

[4] Sattar M.A., Nair A.S., Xavier P.J., Patnaik A. (2019). Natural rubber–SiO_2_ nanohybrids: Interface structures and dynamics. Soft Matter.

[5] Navon G., Shinar H., Eliav U., Seo Y. (2001). Multiple quantum filters and order in tissues. NMR Biomed..

[6] Collignon J., Sillescu H., Spiess H.W. (1981). Pseudo-solid echoes of proton and deuteron NMR in polyethvlene melts. Colloid Polym. Sci..

[7] Callaghan P.T., Samulski E.T. (1997). Molecular Ordering and the Direct Measurement of Weak Proton−Proton Dipolar Interactions in a Rubber Network. Macromolecules.

[8] Kimmich R. (1997). NMR: Tomography, Diffusometry, Relaxometry.

[9] Fechete R., Demco D.E., Blümich B. (2003). Chain Orientation and Slow Dynamics in Elastomers by Mixed Magic-Hahn Echo Decays. J. Chem. Phys..

[10] Demco D.E., Hafner S., Fülber C., Graf R., Spiess H.W. (1996). Two-dimensional proton magnetization-exchange NMR spectroscopy in cross-linked elastomers. J. Chem. Phys..

[11] Sotta P., Fülber C., Demco D.E., Blümich B., Spiess H.W. (1996). Effect of Residual Dipolar Interactions on the NMR Relaxation in Cross-Linked Elastomers. Macromolecules.

[12] Schneider M., Gasper L., Demco D.E., Blümich B. (1999). Residual dipolar couplings by 1H dipolar-encoded longitudinal magnetization, double- and triple-quantum nuclear magnetic resonance in cross-linked elastomers. J. Chem. Phys..

[13] Wang M., Bertmer M., Demco D.E., Blümich B., Litvinov V.M., Barthel H. (2003). Indication of heterogeneity in chain-segment order of a PDMS layer grafted onto a silica surface by ^1^H multiple-quantum NMR. Macromolecules.

[14] Maxwell R.S., Balazs B. (2002). Residual dipolar coupling for the assessment of cross link density changes in gamma irradiated silica PDMS composite materials. J. Chem. Phys..

[15] Saalwächter K., Ziegler P., Spyckerelle O., Haidar B., Vidal A., Sommer J.-U. (2003). ^1^H multiple-quantum nuclear magnetic resonance investigations of molecular order distributions in poly(dimethylsiloxane) networks: Evidence for a linear mixing law in bimodal systems. J. Chem. Phys..

[16] Fechete R., Demco D.E., Blümich B. (2002). Segmental Anisotropy in Strained Elastomers by ^1^H NMR of Multipolar Spin States. Macromolecules.

[17] Saalwächter K. (2007). Proton multiple-quantum NMR for the study of chain dynamics and structural constraints in polymeric soft materials. Prog. Nucl. Magn. Reson. Spectrosc..

[18] Wiesmath A., Filip C., Demco D.E., Blümich B. (2002). NMR of multipolar spin states excitated in strongly inhomogeneous magnetic fields. J. Magn. Reson..

[19] Wiesmath A., Filip C., Demco D.E., Blümich B. (2001). Double-quantum-filtered NMR signals in inhomogeneous magnetic fields. J. Magn. Reson..

[20] Demco D.E., Fechete R., Blümich B. (2003). Residual van Vleck moments in elastomers by accordion magic sandwich. Chem. Phys. Lett..

[21] Malveau C., Tekely P., Canet D. (1997). Visualization of residual anisotropic interactions in crosslinked natural rubbers by dipolar local field measurements and ^2^H natural abundance NMR spectroscopy. Solid State Nucl. Magn. Reson..

[22] Fritzhanns T., Demco D.E., Hafner S., Spiess H.W. (1999). Multi-dimensional ^1^H NMR nuclear Overhauser spectroscopy under magic angle spinning: Theory and application to elastomers. Mol. Phys..

[23] Graf R., Demco D.E., Hafner S., Spiess H.W. (1998). Selective residual dipolar couplings in cross-linked elastomers by ^1^H double-quantum NMR spectroscopy. Solid State Nucl. Magn. Reson..

[24] Bertmer M., Wang M., Demco D.E., Blümich B. (2006). Segmental mobility in short-chain grafted-PDMS by homo- and heteronuclear residual dipolar couplings. Solid State Nucl. Magn. Reson..

[25] Kitaura T., Kobayashi M., Tarachiwin L., Kum-ourm H. (2018). Characterization of Natural Rubber End Groups Using High-Sensitivity NMR. Macromol. Chem. Phys..

[26] Moldovan D., Fechete R., Demco D.E., Culea E., Blümich B., Herrmann V., Heinz M. (2010). Heterogeneity of Nanofilled EPDM Elastomers Investigated by Inverse Laplace Transform ^1^H NMR Relaxometry and Rheometry. Macromol. Chem. Phys..

[27] Moldovan D., Fechete R., Demco D.E., Culea E., Blümich B., Herrmann V., Heinz M. (2011). The heterogeneity of segmental dynamics of filled EPDM by ^1^H transverse relaxation NMR. J. Magn. Reson..

[28] Maxwell R.S., Chinn S.C., Solyom D., Cohenom R. (2005). Radiation-Induced Cross-Linking in a Silica-Filled Silicone Elastomer As Investigated by Multiple Quantum ^1^H NMR. Macromolecules.

[29] Giuliani J.R., Gjersing E.L., Chinn S.C., Jones T.V., Wilson T.S., Alviso C.T., Herberg J.L., Pearson M.A., Maxwell R.S. (2007). Thermal degradation in a trimodal poly(dimethylsiloxane) network studied by ^1^H multiple quantum NMR. J. Phys. Chem. B.

[30] Voda M.A., Demco D.E., Perlo J., Orza R.A., Blümich B. (2005). Multispin moments edited by multiple-quantum NMR: Application to elastomers. J. Magn. Reson..

[31] Venkataramanan L., Song Y.Q., Hurlimann M.D. (2002). Solving Fredholm integrals of the first kind with tensor product structure in 2 and 2.5 dimensions. IEEE Trans. Sign. Proc..

[32] Song Y.Q., Venkataramanan L., Hürlimann M.D., Flaum M., Frulla P., Straley C. (2002). T_1_ − T_2_ correlation spectra obtained using a fast two-dimensional Laplace inversion. J. Magn. Reson..

[33] Callaghan P.T. (2011). Translational Dynamics & Magnetic Resonance.

[34] Nie S., Lacayo-Pineda J., Willenbacher N., Wilhelm M. (2019). Aging of natural rubber studied via Fourier-transform rheology and double quantum NMR to correlate local chain dynamics with macroscopic mechanical response. Polymer.

[35] Huang C., Huang G., Li S., Luo M., Liu H., Fu X., Qu W., Xie Z., Wu J. (2018). Research on architecture and composition of natural network in natural rubber. Polymer.

[36] Munaro A.P., da Chunda G.P., Filgueiras J.G., Pinto J.M., Munaro M., de Azevedo E.R., Akcelrud L.C. (2019). Ageing and structural changes in PDMS rubber investigated by time domain NMR. Polym. Degrad. Stabil..

[37] Fechete R., Demco D.E., Blümich B. (2004). Enhanced sensitivity to residual dipolar couplings by high-order multiple-quantum NMR. J. Magn. Reson..

[38] Hailu K., Fechete R., Demco D.E., Blümich B. (2002). Segmental Anisotropy Induced in Strained Elastomers Detected with a Portable NMR Scanner. Solid state Nucl. Magn. Reson..

[39] Chelcea R.I., Culea E., Demco D.E., Fechete R. (2009). Distributions of transverse relaxation times for soft-solids measured in strongly inhomogeneous magnetic fields. J. Magn. Reson..

[40] Gjersing E., Chinn S., Giuliani J.R., Herberg J., Maxwell R.S., Eastwood E., Bowen D., Stephens T. (2007). Investigation Of Network Heterogeneities In Endlinked, Nonstocihimmetric Reacted PDMS Networks By ^1^H Multiple Quantum NMR. Macromolecules.

[41] Naumova A., Tschierske C., Saalwächter K. (2017). Orientation-dependent proton double-quantum NMR build-up function for soft materials with anisotropic mobility. Solid state Nucl. Magn. Reson..

[42] Chassé W., Valentín J.L., Genesky G.D., Cohen C., Saalwächter K. (2011). Precise dipolar coupling constant distribution analysis in proton multiple-quantum NMR of elastomers. J. Chem. Phys..

[43] Weese J. (1992). A reliable and fast method for the solution of Fredhol integral equations of the first kind based on Tikhonov regularization. Comp. Phys. Commun..

[44] Fechete R., Morar I.A., Moldovan D., Chelcea R.I., Crainic R., Nicoară S.C. (2021). Fourier and Laplace-like low-field NMR spectroscopy: The perspectives of multivariate and artificial neural networks analyses. J. Magn. Reson..

[45] Howse S., Porter C., Mengistu T., Petrov I., Pazur R.J. (2019). Experimental determination of the quantity and distribution of chemical crosslinks in unaged and aged natural rubber. II: A sulfur donor system. Rubber Chem. Technol..

