# Supplementary material for: Bimodal 1H Double Quantum Build-Up Curves by Fourier and Laplace-like Transforms on Aged Cross-Linked Natural Rubber
Source: Polymers (Basel). 2021 Oct 13;13(20):3523. doi: 10.3390/polym13203523 (PMC8540486; doi:10.3390/polym13203523)
Supplement: Supplementary file 1 [file polymers-13-03523-s001.zip › polymers-1383456-supplementary-update.pdf]

Supplementary Materials

Bimodal ^{1}H Double Quantum build-up curves by Fourier and Laplace-like transforms on aged cross-linked natural rubber

D. Moldovan[1*] and R. Fechete[1*]
[1]*Technical University of Cluj-Napoca, Memorandumului 28, R-400114, Cluj-Napoca, Romania,*

A1. The efficiency of various types of DQ pulse sequence for natural rubber

During years the efficiency of creating the double quantum coherences was under debate and various DQ pulse sequences were developed following the general scheme for excitation of DQ coherences with a z-filter as it is shown in SI.1a [1]. The simplest pulse sequence intensively used for the excitation of DQ coherences is based on 5 pulses with a well-known phase cycling. This scheme is presented in Fig. SI.1b, and was used during many years in high and low field to acquire the normalized DQ signal, and in particular was used by us to evaluate the effect of natural aging of NR rubber. A more efficient DQ pulse sequence in

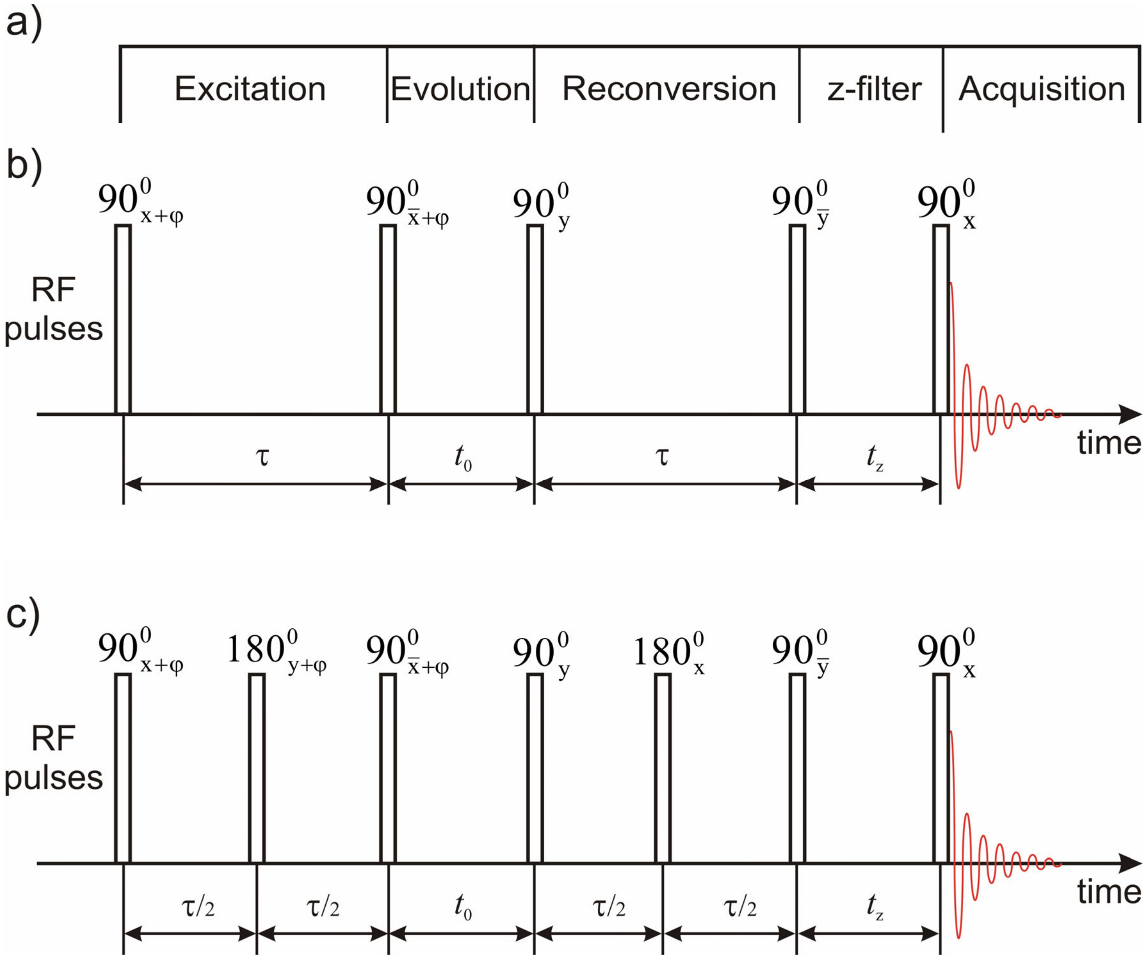

Figure S1. a) General set-up of a double quantum (DQ) NMR experiment; b) five pulses DQ experiment and c) seven pulses DQ experiment with two refocusing pulses in the middle of excitation and reconversion periods.

low and inhomogeneous magnetic field, as in the case of NMR-MOUSE [2], in the middle of excitation and reconversion period a 180° pulse was introduced. The DQ with 7 pulses is presented in Fig. SI.1c. The efficiency of generating the DQ build-up curve was compared for both DQ pulse sequences (e.g. with 5 and 7 pulses) and the results are presented in Fig. SI.2. The main conclusion from these measurement is that for the aged natural rubber the simplest 5 pulse DQ pulse sequence is approximately double in efficiency to generate the DQ coherences. This result is encouraging since, as the pulse sequence is simpler, the theoretical expression of NMR signal is simplest. In the same time, the normalized (to the single quantum signal) DQ signal for six years aged NR1 is at ~ 8.55 %, but for NR7 this efficiency

is increased at ~ 15.4 % for six years aged NR7. In conclusion, one can say that the 5 pulses DQ pulse sequence is efficient in generating DQ coherences.

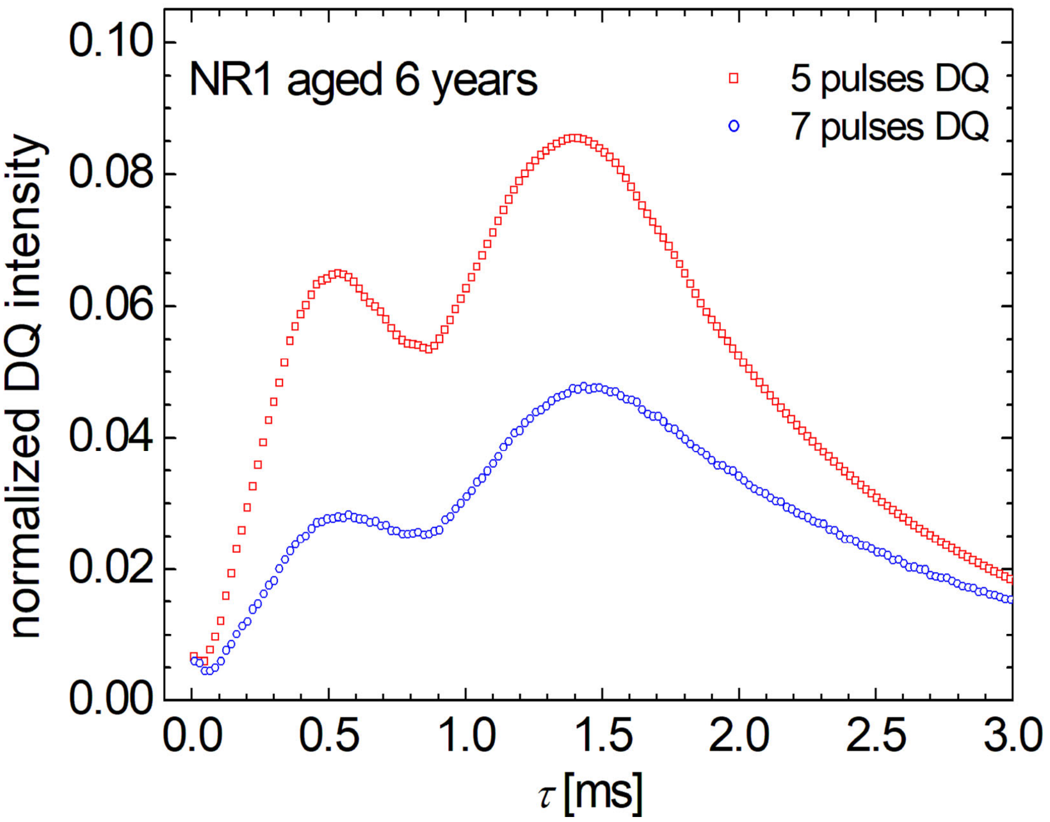

Figure S2. Double quantum (DQ) build-up curves measured for a 6 years natural aged NR1 sample using the 5 pulse DQ sequence (open red square) and 7 pulse DQ sequence (open blue circle).

A2. The influence of azimuthally angle β and R mediation on the residual dipolar coupling distribution

The residual dipolar Hamiltonian which describes a polymer chain between two cross-link points is given by [2-9],

$$\overline{\mathcal{H}}_{\mathrm{D}} = \varpi_D T_{2,0} = \frac{-\sqrt{6}}{2}\overline{D}S_s \frac{1}{Na^2}\left(\vec{R}\right)^2 P_2\left(\cos\beta\right)T_{2,0}, \qquad \text{(S1)}$$

where $S \equiv \overline{P_2\left[\cos\beta^{ij}(t)\right]}$ is the dynamic order parameter, scaled dynamic order parameter is $S_s \equiv S/N_e$, and the instantaneous angle between a given inter-nuclear vector $\vec{r}_{ij}$ and end-to-end vector $\vec{R}$ is $\beta^{ij}(t)$, while β is the angle between the external magnetic field $\vec{B}_0$

and $\vec{R}$. All these quantities were discussed previously, in Ref. [3]. The residual dipolar coupling ϖ_{D} is given by the prefactor to the irreducible tensor operator $T_{2,0}$ in Eq. (SI.1). The double-quantum edited second van Vleck moment of the build-up curve is given by,

$$\overline{M}_2^{DQ} = -\frac{Tr\left\{\left[\overline{H}_D I_z\right]^2\right\}}{Tr\left\{I_z^2\right\}}. \tag{S2}$$

In order to consider the mediation over azimuthally angle β and end-to-end vector $\vec{R}$ mediation on the residual dipolar coupling distribution a statistical average has to be considered. For example, in the case of squared end-to-end vector $\vec{R}$, this is denoted by $\langle(\ldots)\rangle_{\mathrm{R}}$ and is given by,

$$\langle\vec{R}^2\rangle_R \approx Na^2. \tag{S3}$$

where a is the length of a statistical segment and the effective number of statistical segments N is defined by the number of segments $N^{(0)}$ between physical cross links or topological constraints and the number $N^{(C)}$ of segments between chemical cross-links. With these notation the residual dipolar Hamiltonian becomes,

$$\overline{\mathcal{H}}_{\mathrm{D}} = \frac{-\sqrt{6}}{2}\frac{\vec{R}^2}{\left\langle\vec{R}^2\right\rangle_R}\mathrm{P}_2(\cos\beta)\overline{D}S_s(\mathrm{N})\mathrm{T}_{2,0}, \tag{S4}$$

where, in general the scaled dynamic parameter depends on the number of statistical segments $S_s(\mathrm{N})$. The statistical average $\langle(\ldots)\rangle$ from Eq. (3) can be written extensively as,

$$\langle(\ldots)\rangle = \left\langle\left\langle\langle(\ldots)\rangle_\gamma\right\rangle_\beta\right\rangle_{\vec{\mathrm{R}}} \tag{S5}$$

Then, for a given polymeric network the DQ signal is,

$$\begin{aligned} S_{DQ}\left(2\tau, T_2^*\right) &= \left\langle \sin^2(\overline{\omega}_{\mathrm{D}}\tau)\exp\left(-\frac{2\tau}{T_2^*}\right)\right\rangle = \exp\left(-\frac{2\tau}{T_2^*}\right)\left\langle\left\langle\left\langle\sin^2\left[\overline{\omega}_D\left(\vec{R},\beta\right)\cdot\tau\right]\right\rangle_\gamma\right\rangle_\beta\right\rangle_{\vec{\mathrm{R}}}, \\ &= \exp\left(-\frac{2\tau}{T_2^*}\right)\int_0^\infty\int_0^\pi\int_0^{2\pi} g\left(\vec{R}\right)\cdot h(\beta)\cdot l(\gamma)\sin^2\left[\overline{\omega}_D\left(\vec{R},\beta\right)\cdot\tau\right]d\gamma d\beta\cdot dR \end{aligned} \tag{S6}$$

in general, where we have not considered the distribution over T_2^* and number of statistical segments. In particular, by assuming an isotropic sample and a Gaussian distribution of the end-to-end vector $g\left(\vec{R}\right)$, the DQ signal can be rewritten as,

$$S_{DQ}\left(2\tau,T_2^*\right) = \exp\left(-\frac{2\tau}{T_2^*}\right)\int_0^{\infty}\frac{\vec{R}^2}{\pi}\left(\frac{3}{2\pi\left\langle\vec{R}^2\right\rangle_{\vec{R}}}\right)^{3/2}\exp\left[-\frac{3R^2}{2\left\langle\vec{R}^2\right\rangle_{\vec{R}}}\right]\cdot\int_0^{\pi}\sin(\beta)\sin^2\left[\overline{\omega}_D(R,\beta)\cdot\tau\right]d\beta\cdot dR, \tag{S7}$$

since $h(\beta)=\sin(\beta)$ and $l(\gamma)=1$ due to the isotropy.

The main question is to describe the relation between the distribution of the residual dipolar coupling $f\left(\overline{\omega}_{\mathrm{D}}\right)$ from eq. (18) and the physical quantities which defines the dipolar Hamiltonian from eq. (SI.4). For that, first we will introduce new quantities like the residual dipolar coupling RDC as,

$$D_{res} = \frac{-3}{2}\overline{D}S_s(\mathrm{N}), \tag{S8}$$

and the dimensionless squared end-to-end vector,

$$\vec{q}^{\,2} = \frac{\vec{R}^2}{\left\langle\vec{R}^2\right\rangle_R} = \frac{\vec{R}^2}{Na^2}, \tag{S9}$$

then we can write,

$$\overline{\omega}_{\mathrm{D}}\left(\vec{R},\beta,D_{res}\right) = \vec{q}^{\,2}D_{res}\mathrm{P}_2(\cos\beta) \tag{S10}$$

and the DQ signal become,

$$S_{DQ}\left(2\tau,T_2^*\right) = \left(\frac{3}{2\pi^{5/3}}\right)^{3/2}\exp\left(-\frac{2\tau}{T_2^*}\right)\times \times\int_0^{\infty}P\left(D_{res}\right)\int_0^{\infty}\vec{q}^{\,2}\exp\left[-\frac{3}{2}\vec{q}^{\,2}\right]\cdot\int_0^{\pi}\sin(\beta)\sin^2\left[\vec{q}^{\,2}\mathrm{P}_2(\cos\beta)D_{res}\cdot\tau\right]d\beta\cdot dq\cdot dD_{res}, \tag{S11}$$

In this point one would like to test the effect each function in the expression of residual dipolar coupling $\overline{\omega}_D$ from eq. SI.10. For that we implemented a program written in C++ to generate a large number of random numbers with a specific distribution. In order to do that, the Metropolis algorithm was implemented [10]. Thus, for the first test, the sinusoidal distribution of azimuthally angle β, between the limits of 0 and π (see the insert in Fig. SI.3a) was used to characterize the distribution of $\overline{\omega}_D$ assuming a constant product $\vec{q}^{\,2}D_{res}$. The result is presented in Fig. SI.3a. The results may be better validated presenting also the symmetric representation of the shapes of the resulted distribution in respect to the origin

point (see Fig. SI.3b). Now one can recognize the well know Pake doublet, or the powder average [11].

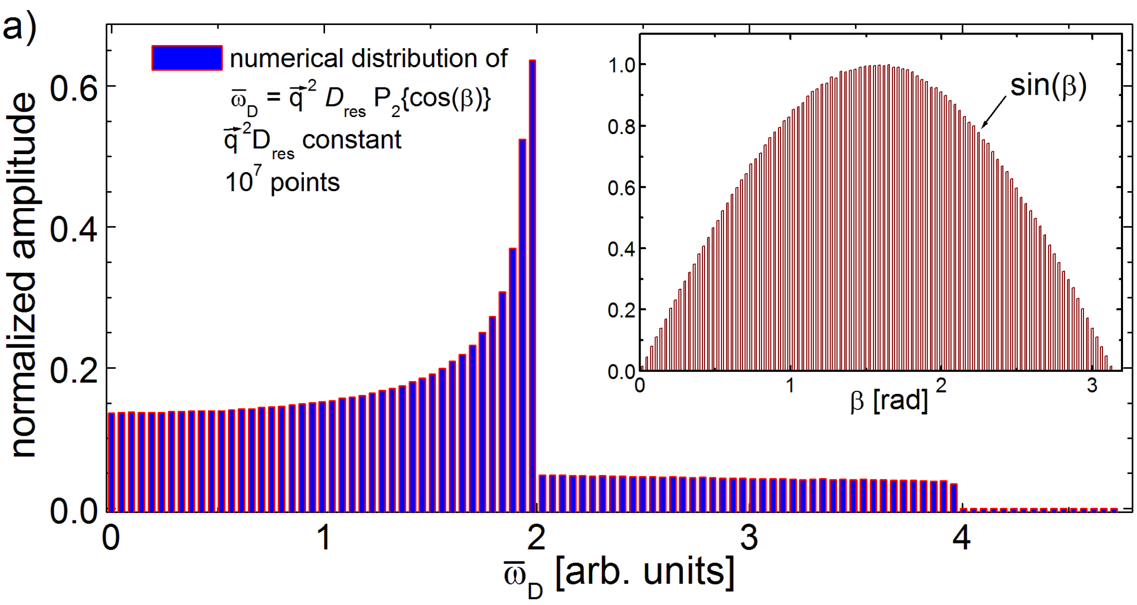

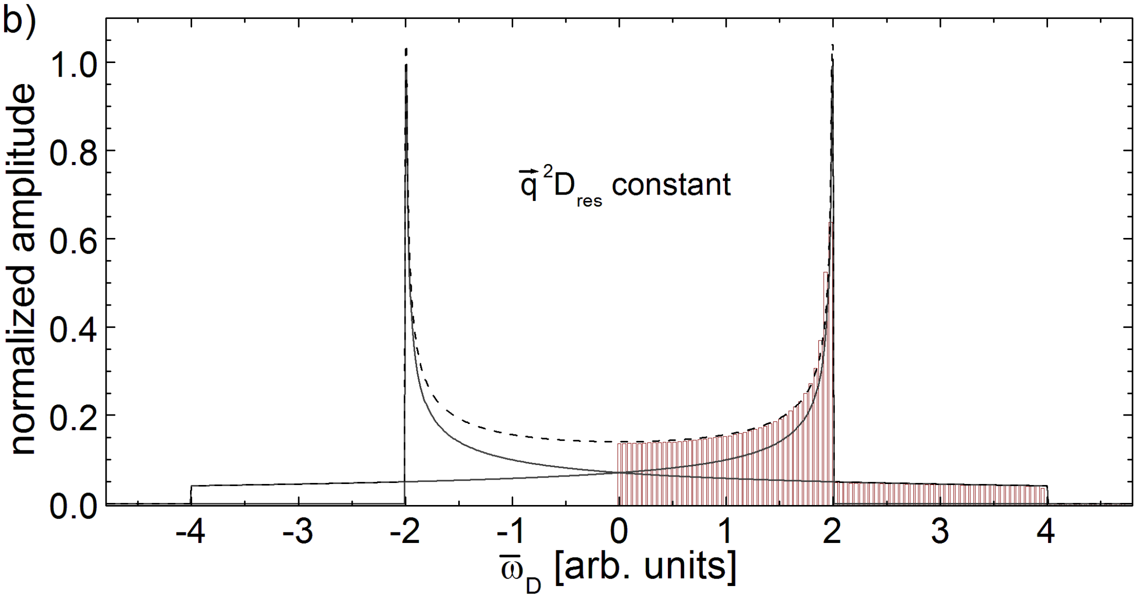

Figure S3. a) The simulated bar plot distribution of $\overline{\omega}_D$ given by the equation (SI.10) for constant $\vec{q}^2 D_{res}$ considering an isotope angular dependence described by *sin* function of azimuthal angle β shown in the small inclusion; b) the comparison between the positive simulated bar plot distribution of $\overline{\omega}_D$ from Fig. a) and a specific powder spectra.

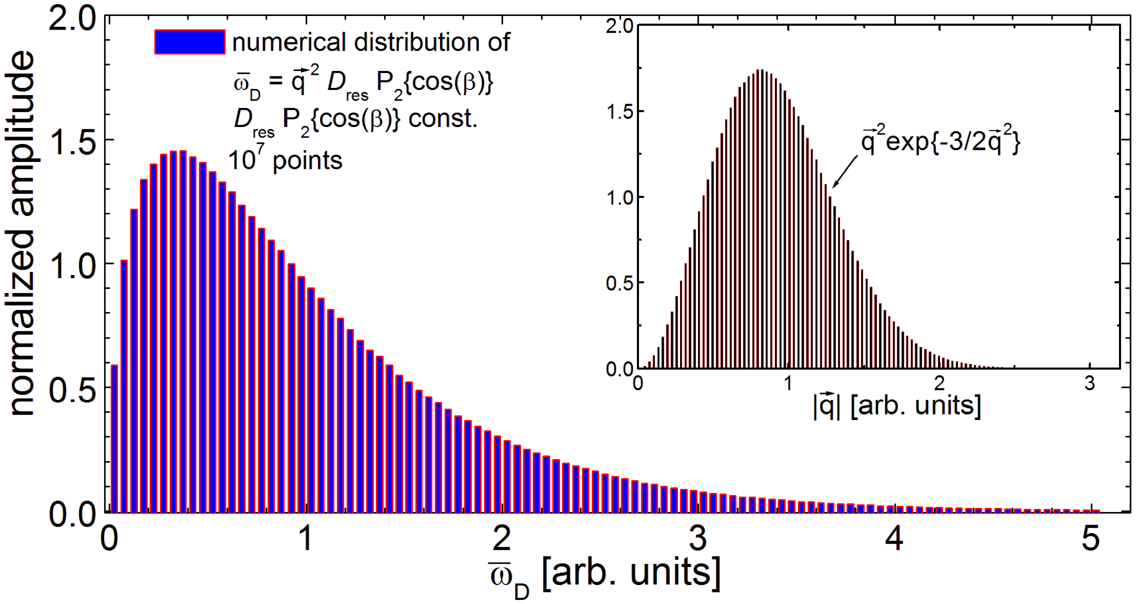

Figure S4. The simulated bar plot distribution of $\overline{\omega}_D$ given by the equation (SI.9) for constant $D_{res}P_2\{\cos(\beta)\}$ considering only the distribution of dimensionless squared end-to-end vector shown in the small insert.

In the next step a Gaussian distribution of the dimensionless squared end-to-end vector, $\vec{q}^2$ was assumed as it is presented in the insert of the figure SI.4. In this case the product $D_{res}P_2\{cos(\beta)\}$ was considered constant and the resulted distribution of $\overline{\omega}_D$ is presented in figure SI.4. The shape of the simulated distribution of $\overline{\omega}_D$ is again known to be the Γ function defined as $P_\Gamma(\overline{\omega}_D)$ in eq. 14. As it can be observed, the Γ distribution of residual dipolar coupling $\overline{\omega}_D$ is broad and presents a significant contribution at small $\overline{\omega}_D$ values. With these two tests the validity of the Metropolis algorithm was demonstrated by obtaining the expected results. In conclusion, the tested Metropolis algorithm can be used to find out the distribution of a relevant quantity if we know the distribution function of all parameters that describes that quantity.

Next, both distributions, over the azimuthally angle β and over the squared end-to-end vector, $\vec{q}^2$ were assumed simultaneously. The result is presented in Fig. SI.5a for a constant value of the residual dipolar coupling D_{res}. A surprising result come out from the simulations where the residual dipolar coupling $\overline{\omega}_D$ presents the largest probability at zero value and

decay (exponentially) with the increase of the value of $\overline{\omega}_D$. This kind of distribution of $\overline{\omega}_D$ was not observed experimentally. This leads to the conclusion that the residual dipolar coupling constant D_{res} himself is characterized by a certain distribution or/and the assumed functions for the azimuthally angle β or dimensionless squared end-to-end vector, $\vec{q}^2$ are not

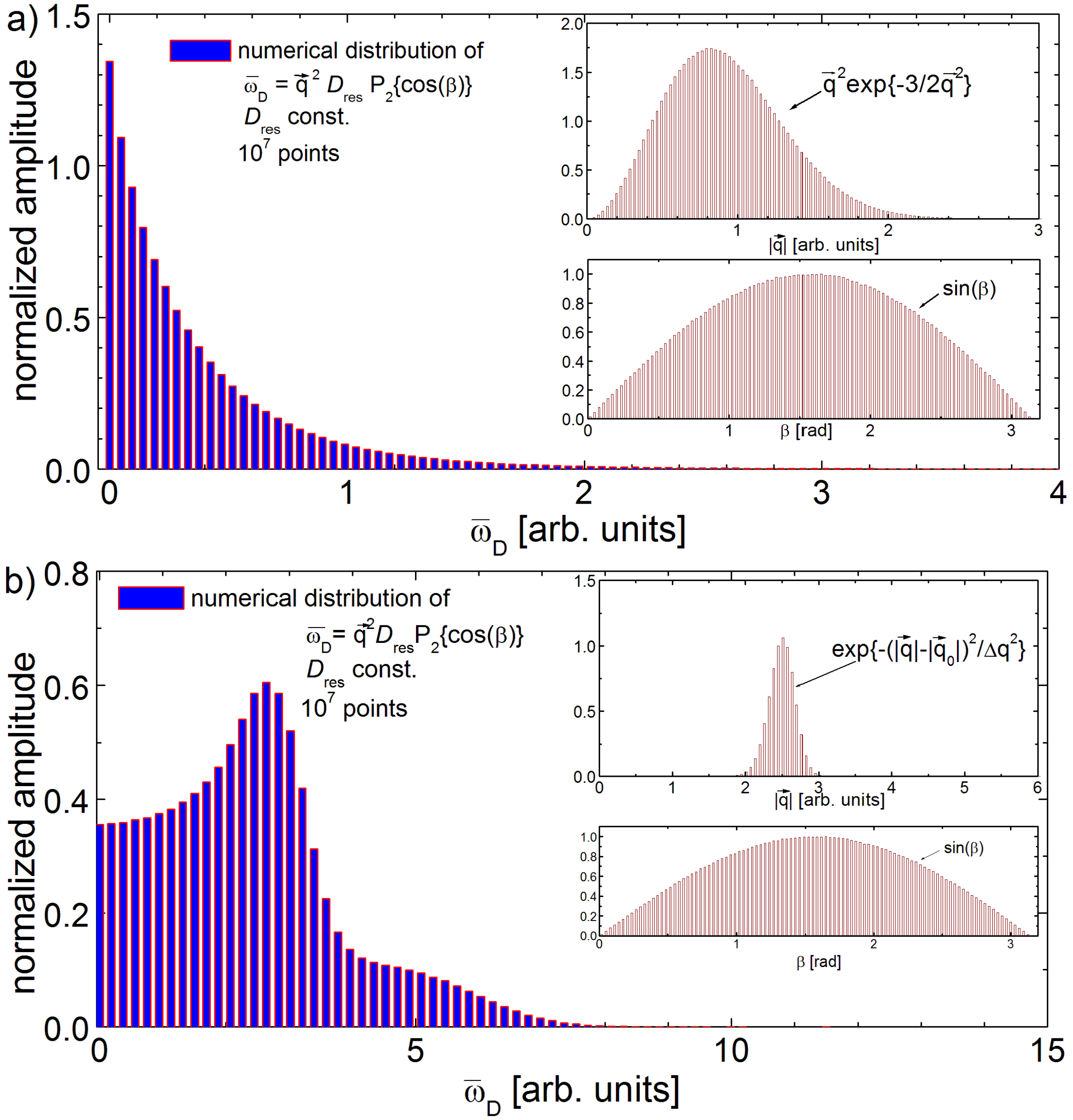

Figure S5. The simulated bar plot distribution of $\overline{\omega}_D$ given by the equation (SI.10) for constant D_{res} for a combined isotope angular dependence described by *sin* function of azimuthal angle β and a) a 3D Gaussian distribution of the end-to-end vector and b) an Gaussian distribution of dimensionless squared end-to-end vector with the center $|\vec{q}_0| = 2.5$.

distributed as it was assumed. The orientation isotropy cannot be questioned even for the aged natural rubber, therefore once can test a change in the distribution of squared end-to-end vector. This assumes that the dimensionless squared end-to-end vector is no longer centered in 0 but will have a new center $|\vec{q}_0| = 2.5$ (in our simulation). In the same time the new distribution of squared end-to-end vector, $\vec{q}^2$ is narrowed and the width is described by the factor Δq. The results of this simulation is presented in Fig. SI.5b. Now the distribution of residual dipolar coupling $\bar{\omega}_D$ is more similarly to that measured for NR1 naturally aged for 6 years, with the exception of probabilities shown at small $\bar{\omega}_D$ values. In this point we have no physical argument to consider that such a shift of dimensionless squared end-to-end vector, $\vec{q}^2$ is happening in the aged natural rubber, therefore our focus will be then oriented to the distribution of residual dipolar coupling constant D_{res}.

A3. The distribution of residual dipolar coupling constant D_{res}

In order to obtain the distribution of the residual dipolar coupling constant D_{res} the double quantum NMR signal can be rewritten as,

$$S_{DQ}\left(2\tau, T_2^*\right) = \frac{1}{\pi}\exp\left(-\frac{2\tau}{T_2^*}\right)\int_0^\infty P\left(D_{res}\right)K\left(D_{res}\right)\cdot dD_{res}\ , \tag{S12}$$

where the kernel, consider the mediation over dimensionless squared end-to-end vector, $\vec{q}^2$ and azimuthal angle β, and it is given by:

$$\begin{aligned} K\left(D_{res}\right) &= \left\langle\left\langle \sin^2\left[\vec{q}^2 \mathrm{P}_2\left(\cos\beta\right)D_{res}\tau\right]\right\rangle_q\right\rangle_\beta \\ &= \left(\frac{3}{2\pi}\right)^{3/2}\int_0^\infty \vec{q}^2 \exp\left[-\frac{3}{2}\vec{q}^2\right]\cdot\int_0^\pi \sin\left(\beta\right)\sin^2\left[\vec{q}^2\mathrm{P}_2\left(\cos\beta\right)D_{res}\cdot\tau\right]d\beta\cdot dq \end{aligned}\ , \tag{S13}$$

The residual dipolar coupling constant D_{res}, more specific the distribution of D_{res} is a specific feature of each sample (in our case of aged natural rubber). In the first step we will consider, that the distribution of D_{res} is of Gaussian form and it is centered to a specific value

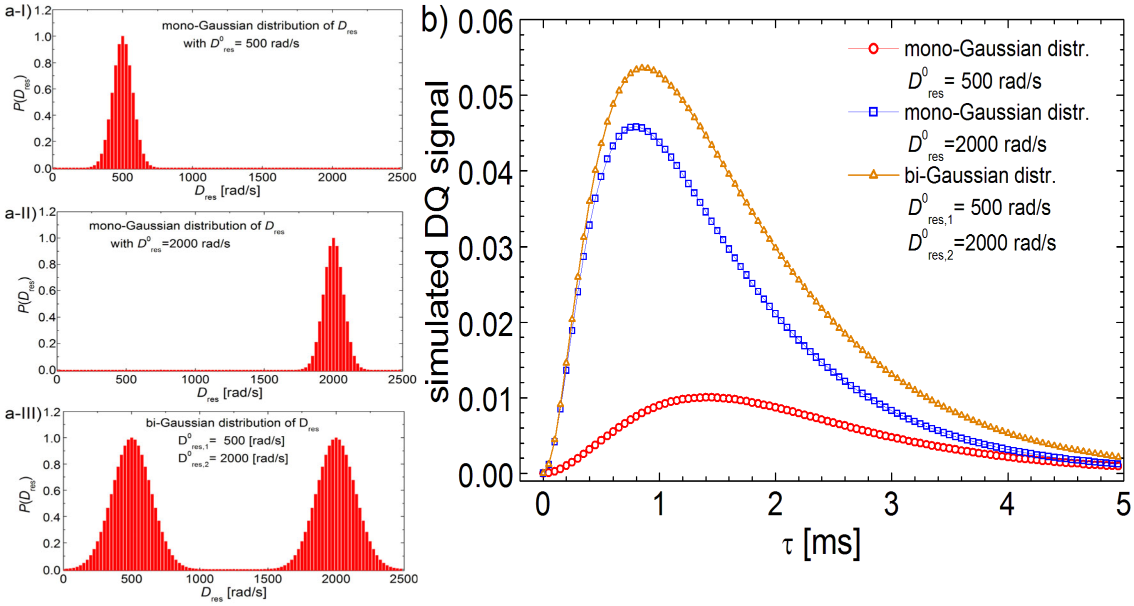

Figure S6. a) Simulated bar plot distributions of residual dipolar coupling constant $P(D_{res})$ for a: I) mono-Gaussian with $D^0_{res} = 500\, rad/s$; II) mono-Gaussian with $D^0_{res} = 2000\, rad/s$ and III) bi-Gaussian with $D^0_{res,1} = 500\, rad/s$ and $D^0_{res,2} = 2000\, rad/s$. b) The simulated DQ build-up curves using eq. (SI.12) and kernel (SI.13) for the simulated distributions presented in a) and $T_2^* = 2$ ms.

D^0_{res}. In figure SI.6 a) are presented a series of simulation of a mono-Gaussian distributions of D_{res} with $D^0_{res,1} = 500\, rad/s$ (Fig. SI.6 a-I) and with $D^0_{res,2} = 2000\, rad/s$ (Fig. SI.6 a-II). The sum of these two distribution can be considered as a bi-Gaussian distributions of D_{res} and it is presented in Fig. SI.6 a-III. The simulated DQ build-up curves using eq. SI.12 for the DQ signal and eq. SI.13 for the kernel are presented in Fig. SI.6 b for all three distributions of $P(D_{res})$. Thus, with open circles is plotted the DQ-NMR signal for the mono-Gaussian distribution of residual dipolar coupling constant $P(D_{res})$ with $D^0_{res,1} = 500\, rad/s$ (shown in Fig. SI.6 a-I), with open squares is plotted the DQ-NMR signal for $P(D_{res})$ with $D^0_{res,2} = 2000\, rad/s$ (shown in Fig. SI.6 a-II) and with open triangles is plotted the DQ build-

up curve for the bi-Gaussian distribution of $P(D_{res})$ shown in SI.6 a-III). In all cases the effective relaxation time T_2^* was 2 ms.

As expected, comparing the DQ build-up curves simulated for a mono-Gaussian distribution of residual dipolar coupling constant D_{res}, the slope of DQ-NMR signal in the initial time regime reflects the value (center value) of D_{res}. Nevertheless, the simulated DQ build-up curve for the bi-Gaussian distribution of residual dipolar coupling constant D_{res} does not present the expected two components as was observed for example in the case of the measured DQ build-up curve for six years aged NR1. Therefore, one can assume that the eq. SI.12 with SI.13 kernel having as a result a specific distribution of residual dipolar coupling constant D_{res} cannot (satisfactory) explain the measured data.

In the attempt to generate the shapes of the DQ build-up curves for the measured aged natural rubber with different cross-link density one can assume one more changes to the DQ build-up curve. The change assumes that each distribution is characterized by another effective relaxation time, and the DQ-NMR signal can be rewritten as,

$$\begin{aligned} S_{DQ}(2\tau, T_2^*) = \frac{1}{\pi} exp\left(-\frac{2\tau}{T_{2,1}^*}\right) \cdot \int_{D_{res,1}^{min}}^{D_{res,1}^{max}} P(D_{res})\, K(D_{res}) \cdot dD_{res} + \\ + \frac{1}{\pi} exp\left(-\frac{2\tau}{T_{2,2}^*}\right) \cdot \int_{D_{res,2}^{min}}^{D_{res,2}^{max}} P(D_{res})\, K(D_{res}) \cdot dD_{res} \end{aligned} \qquad \text{(S14)}$$

where the kernels are given by eq. SI.13 and the effective relaxation times $T_{2,1}^*$ is associated with small D_{res} (centered on $D_{res,1}^0$) values and $T_{2,2}^*$ is associated with large D_{res} values (centered on $D_{res,2}^0$). One can remark that for those two distributions here there are a finite lover and upper limits $D_{res,1(2)}^{min}$ and $D_{res,1(2)}^{max}$, respectively.

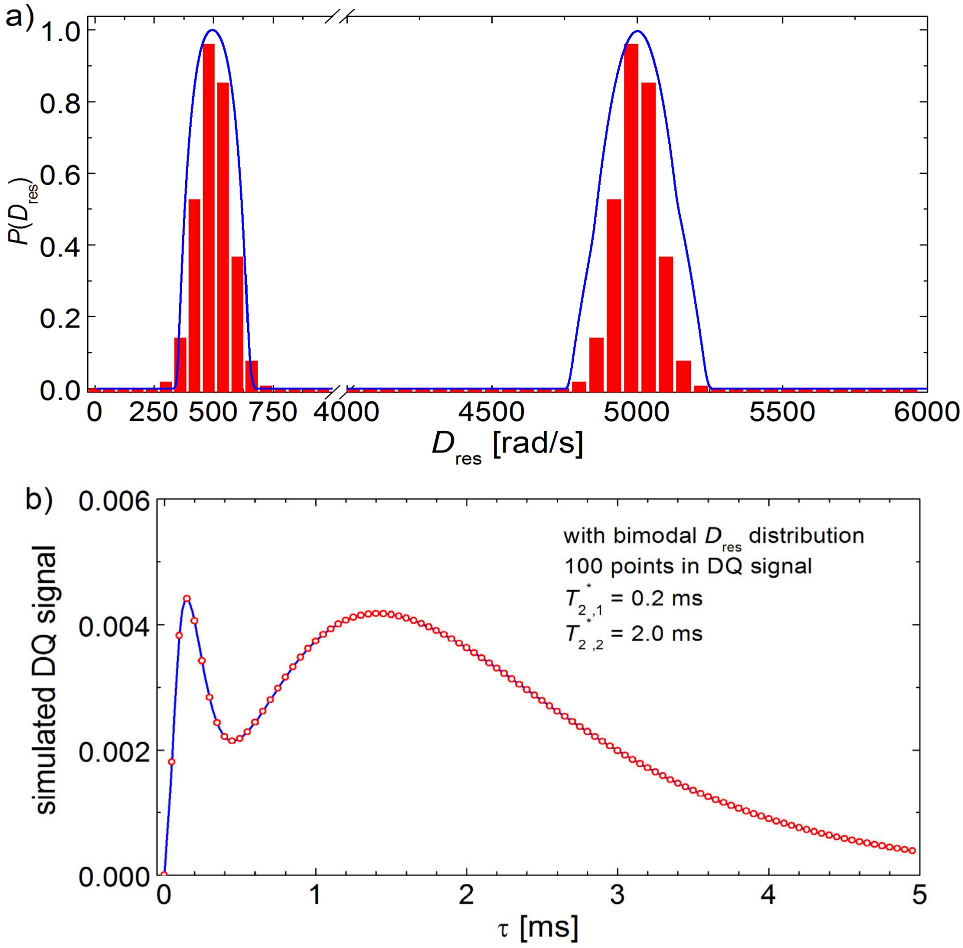

Figure S7. a) Simulated bar plot bi-Gaussian distributions of residual dipolar coupling $P(D_{res})$ with $D^0_{res,1} = 500\, rad/s$ and $D^0_{res,2} = 5000\, rad/s$ and the resulted residual dipolar coupling constant distribution $P(D_{res})$ (continuous line) after Laplace-like analysis based on eq. (SI.14) with $T^*_{2,1} = 2\, ms$ and $T^*_{2,2} = 0.2\, ms$. b) The simulated DQ build-up curve (open circles) using eq. (SI.13) and the fitting of simulated DQ build-up data (continuous line).

In Fig. SI.7 a) a bi-Gaussian distribution of residual dipolar coupling constant D_{res} is presented as red bar plot centered on $D^0_{res,1} = 500\, rad/s$ and $D^0_{res,2} = 5000\, rad/s$ with the same width. These distributions are used in eq. SI.14 to generate the DQ build-up curve with $T^*_{2,1} = 2\, ms$ and $T^*_{2,2} = 0.2\, ms$. The simulated data are represented with open circles. One can observe that in this case we get a DQ build-up curve that presents a shape closely related to the shape of the DQ build-up curve measured for natural aged NR1-NR7 for six years. Nevertheless, in this case the first peak located at lover time values is narrower compared to the peak located at large time value, fact that is not observed for the experimental data.

The distribution obtained by Laplace-like inversion using eq. SI.14 is presented with a continuous blue line on top of the simulated distributions in Fig. SI.7a. Good superposition between those two distributions (generated and obtained by inversion from simulated data) is observed therefore one can conclude that the inversion algorithm works well. Moreover as a result of inversion, the NMR signal (continuous line in Fig. SI.7b) fit well the simulated DQ build-up data (open circles in Fig. SI.7b).

A similarly DQ build up curve vas obtained experimentally by Bertmer et al. [12] for a PDMS1 sample (PDMS content, 44 wt%, and average chain length N_t = 7.7). Similarly data are presented in Fig. SI.8a) and are used to determine the distribution of residual dipolar coupling constant D_{res} using eq. SI.14. The fit of the data are presented in the same figure with dashed line. One can observe a very good approximation of the input data. The resulted distributions characterized by $T_{2,1}^{*} = 0.22\ ms$ (dashed line) associated with the large values of D_{res} and $T_{2,2}^{*} = 3.92\ ms$ (continuous line) associated with the small values of D_{res} are presented in Fig. SI.8b. The values of $T_{2,1}^{*}$ and $T_{2,2}^{*}$ are resulted from fit in the Laplace-like inversion process. As one can observe each distribution of D_{res} consist in two Gauss-like peaks.

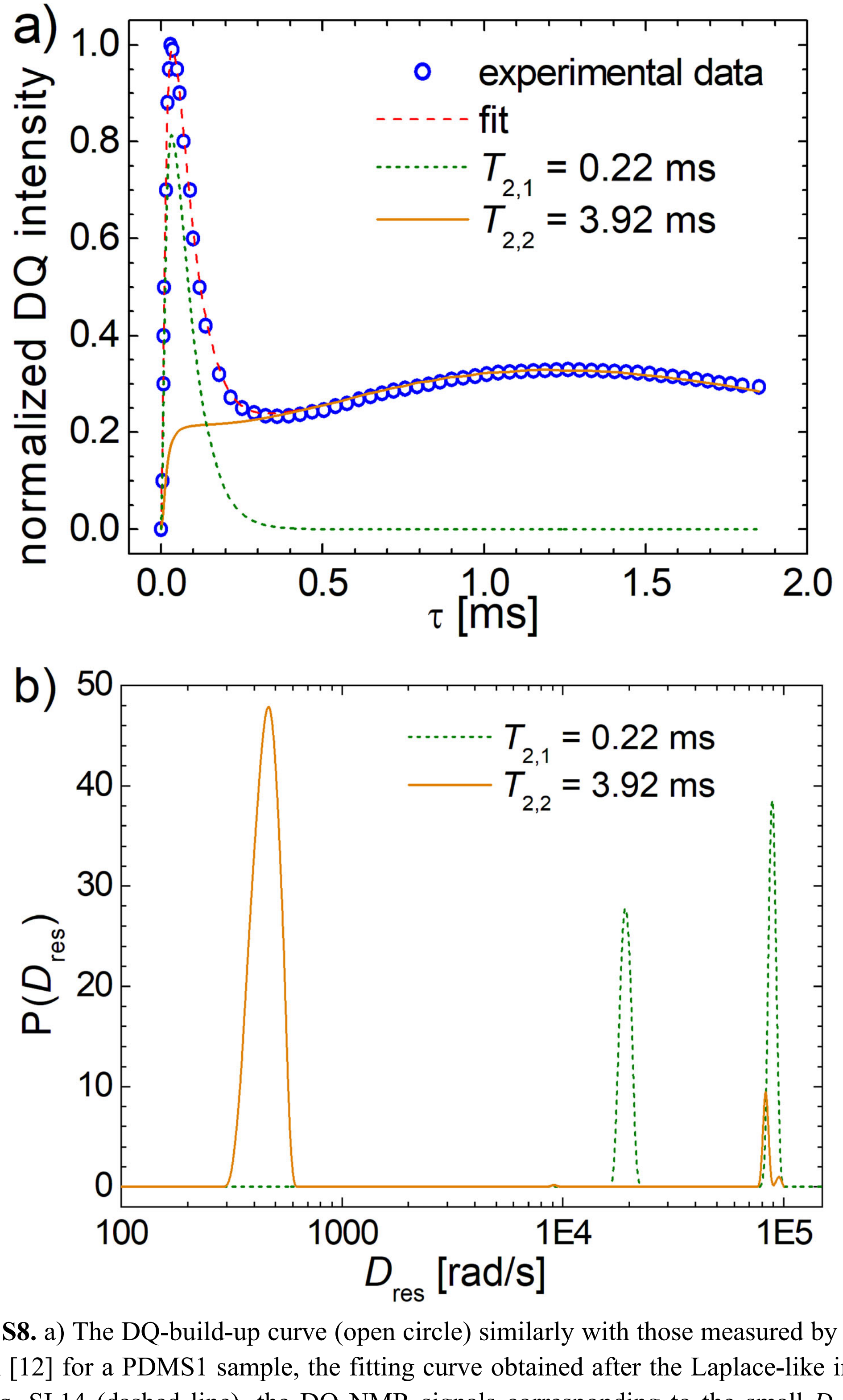

Figure S8. a) The DQ-build-up curve (open circle) similarly with those measured by Bertmer et al. in [12] for a PDMS1 sample, the fitting curve obtained after the Laplace-like inversion using eq. SI.14 (dashed line), the DQ NMR signals corresponding to the small D_{res} values (continuous orange line) and to the large D_{res} values (dotted olive line); b) The distributions of residual dipolar coupling constants resulted from the analysis of data presented in a) by Laplace-like inversion using eq. SI.14 with $T_{2,1}^{*} = 0.22\ ms$ and $T_{2,2}^{*} = 3.92\ ms$.

We are not going to discuss more about this result, but we would like to point out that the equation SI.14 with the kernel described by eq. SI.13 which implies the powder average can be successfully used for a better analysis of some experimental data presenting two components.

The final test of practical ability of eq. SI.14 to be used to obtain the distribution of residual dipolar coupling constant D_{res} is to be applied on the measured data for our series of natural aged NR samples with different cross-link density. We are interested first to see if this approach can fit the experimental data, especially for the NR1 of which DQ build-up curve presents the most pronounced (separated) two peaks. Then one should compare the merit value χ^2 (eq. 9) obtained for the same DQ build-up curve inverted using the eq. 20 to get the distribution of the averaged residual dipolar couplings $\overline{\omega}_D$ with those inverted using eq. SI.14 to get obtain the distribution of residual dipolar coupling constant D_{res}. The goal is to verify if the DQ build-up curve can be fitted better using the complete definition (with the powder average) of the kernel. In this sense the best fit of the DQ build-up curve measured for NR1 natural aged during six years analyses by Laplace-like inversion using eq. SI.11 with the kernel SI.12 is presented in Fig. 8a from the main manuscript. This is far away from the best fit presented in Fig. 9a and analyzed by eq. 18. In conclusion, our experimental data of the cross-linked series of NR rubber six year aged cannot be better analyzed using the mono (eq. SI.12) or bi-modal (eq. SI.14) distributions of the residual dipolar coupling constant D_{res} where the kernel (eq. SI.13) imply the average over dimensionless squared end-to-end vector, $\vec{q}^2$ and azimuthal angle β. One can assume that the demonstrated inability of the previous (completely) described function to describe the DQ build-up curve measured for the natural aged cross-linked NR series is related to the implicit assumptions of the particular distributions of dimensionless squared end-to-end vector, $\vec{q}^2$ and azimuthal angle β. Most probably that, in the case of aged rubber, these distributions of $\vec{q}^2$ and β are affected (into an unknown way) by natural aging.

References

1. Bertmer, M.; Gasper, L.; Demco, D.E.; Blümich, B.; Litvinov, V.M. *Macromol. Chem. Phys.* **2004**, *205*, 83–94.
2. Fechete, R.; Demco, D.E.; Blümich, B. Enhanced sensitivity to residual dipolar couplings by high-order multiple-quantum NMR. *J. Magn. Reson.* **2004**, *169*, 19–26.
3. Fechete, R.; Demco, D.E.; Blümich, B. Chain Orientation and Slow Dynamics in Elastomers by Mixed Magic-Hahn Echo Decays. *J. Chem. Phys.* **2003**, *118*, 2411–2421.
4. Wiesmath, A.; Filip, C.; Demco, D.E.; Blümich, B. Double-quantum-filtered NMR signals in inhomogeneous magnetic fields. *J. Magn. Reson.* **2001**, *149*, 258–263.
5. Voda, M.A.; Demco, D.E.; Perlo, J.; Orza, R.A.; Blümich, B. Multispin moments edited by multiple-quantum NMR: Application to elastomers. J. Magn. Reson. 2005, 172, 98–109.
6. Fechete, R.; Demco, D.E.; Blümich, B. Enhanced sensitivity to residual dipolar couplings by high-order multiple-quantum NMR. *J. Magn. Reson.* **2004**, *169*, 19–26.
7. Hailu, K.; Fechete, R.; Demco, D.E.; Blümich, B. Segmental Anisotropy Induced in Strained Elastomers Detected with a Portable NMR Scanner. *SS NMR* **2002**, *22*, 327–343.
8. Chelcea, R.I.; Culea, E.; Demco, D.E.; Fechete, R. Distributions of transverse relaxation times for soft-solids measured in strongly inhomogeneous magnetic fields. *J. Magn. Reson.* **2009**, *196*, 178–190.
9. Heise, H.; Sakellariou, D.; Meriles, C.A.; Moulé, A.J. Two-dimensional high-resolution NMR spectra in matched B(0) and B(1) field gradients. *J. Magn. Reson.* **2002**, *156*, 146–151.
10. Metropolis, N.; Rosenbluth, A.; Rosenbluth, M.; Teller, A.; Teller, E. Equation of State Calculations by Fast Computing Machines. *J. Chem. Phys*. **1953**, *21*, 1087.
11. Canet, D. *Nuclear Magnetic Resonance. Concept and Methods*; John Wiley & Sons Ltd.: Hoboken, NJ, USA, 1996.
12. Bertmer, M.; Wang, M.; Demco, D.E.; Blümich, B. Segmental mobility in short-chain grafted-PDMS by homo- and heteronuclear residual dipolar couplings. *Solid State Nucl. Magn. Reson.* **2006**, *30*, 45–54.
